# Supplementary material for: Mechanobiological model for simulation of injured cartilage degradation via pro-inflammatory cytokines and mechanical stimulus
Source: PLoS Comput Biol. 2020 Jun 25;16(6):e1007998. doi: 10.1371/journal.pcbi.1007998 (PMC7343184; doi:10.1371/journal.pcbi.1007998)
Supplement: S1 Supplementary Material — The supplementary material containing more detailed information about the underlying experimental findings, biochemical and biomechanical models, finite element mesh density, physiological levels of interleukin-1, and estimations of degraded cartilage area over time. (DOCX) [file pcbi.1007998.s001.docx]

**Electronic Supplementary Material**

For “*Mechanobiological model for simulation of injured cartilage degradation via pro-inflammatory cytokines and mechanical stimulus*” (in *PLOS Computational Biology*) by

Atte S.A. Eskelinen^1^, Petri Tanska^1^, Cristina Florea^1,2^, Gustavo A. Orozco^1^, Petro Julkunen^1,3^, Alan J. Grodzinsky^2^ and Rami K. Korhonen^1^.

^1^*Department of Applied Physics, University of Eastern Finland, Yliopistonranta 1 F, 70211 Kuopio, Finland*

^2^*Departments of Biological Engineering, Electrical Engineering and Computer Science and Mechanical Engineering, Massachusetts Institute of Technology, 77 Massachusetts Avenue, Cambridge, MA 02139, USA*

^3^*Department of Clinical Neurophysiology, Kuopio University Hospital, Puijonlaaksontie 2, 70210 Kuopio, Finland*

**Corresponding author:**

*Atte S.A. Eskelinen

Department of Applied Physics

University of Eastern Finland

Yliopistonranta 1 F

POB 1627, Kuopio FI-70211, Finland

Tel: +358 40 748 3373

[attees@uef.fi](mailto:attees@uef.fi)

**S1 Materials and methods**

**S1.1 Previous experiments**

Tissue harvest, culture, injurious compression and dynamical loading protocol are described in detail in earlier studies [1–6]. Briefly, in previous experiments, the biochemical and biomechanical integrity of skeletally immature bovine cartilage explants were compromised via exogenous cytokine challenge [1,3,5] or mechanical injury followed by dynamic loading [6], respectively. Furthermore, Li et al. [4] included these mechanisms simultaneously with and without mechanical injury. 3-mm-diameter cartilage samples were obtained from patellofemoral grooves of 1-2-week-old calves. The top 1-mm of the samples including the intact superficial zone of cartilage was cut. For location-matching, each treatment group had samples originating from all regions along cartilage surface. The disks were equilibrated in culture for two days (37 °C, 5% CO_2_) in serum-free medium: 1 g/l Dulbecco’s Modified Eagles Medium (DMEM) plus 10% fetal bovine serum (FBS, [6]) or 1% insulin-transferrin-selenium (ITS, [4]), 10 mM HEPES buffer, non-essential amino acids, proline, ascorbate, and penicillin-streptomycin mixture. The culture medium, which was free of endogenous growth factors, was changed every two days.

**S1.2 Experimental biochemical degradation**

Li et al. [5] cultured mechanically intact cartilage disks with or without (*n* = 24 cartilage disks for both) exogenous pro-inflammatory cytokine IL-1α present at a concentration of 1 ng/ml for eight days representing moderate inflammation. They also performed another set of cytokine experiments where the culture medium was analyzed at days 2, 4, 6, 8, 10, 12, 14, 16, 20, and 25 (*n* = 6 disks at each timepoint with and without IL-1α) after initiation of the cytokine challenge to quantify time-dependent GAG loss via DMMB assay. Besides cytokine concentration of 1 ng/ml, also 10 and 100 ng/ml concentrations have been used [1,3,7].

**S1.3 Experimental biomechanical degradation**

To induce chondral lesions, Orozco et al. [6] injured cartilage disks (*n* = 28) with radially unconfined compression (50% strain amplitude with strain rate 100%/s) [8–10]. Ten of these disks served as day 0 injured controls, while the rest were subjected to a dynamic loading protocol (unconfined compression, haversian waveform, 15% axial strain amplitude, 1 Hz frequency, simulating walking) [4,6] for 7 (*n* = 9) or 12 (*n* = 9) days. Location-matched freely-swollen controls (*n* = 10 for day 0, *n* = 11 for day 7, *n* = 9 for day 12) and uninjured dynamically loaded disks (*n* = 10 for day 7, *n* = 11 for day 12) were also analyzed for loss of optical density via digital densitometry (DD [6], 4x magnification, pixel size 1.563 μm). Moreover, FCD losses at days 7 and 12 were calculated as decrease in optical density with respect to day 0 level of optical density.

In the current study, we quantified average FCD loss near lesions and away from them to deepen our knowledge of PG matrix damage when chondral lesions are present, from the data originally presented by Orozco et al. [6]. All the cartilage disks had been cut into three histological slices, and we included them all to our analyses except the ones that had shattered into pieces. In injured disks, average optical density was determined **(1)** within 0.1 mm (±10%) from lesion by selecting lesion edges and using morphological dilation in MATLAB (Image Processing Toolbox), and **(2)** away from lesion from a 0.15 mm deep (±10%, from the surface) and 0.45 mm wide (±10%) region located at the middle between lesion and disk edges. For uninjured samples, the optical density was calculated from a 0.15 mm deep (±10%) and 0.45 mm wide (±10%) region located at the middle of disk. We chose these region sizes according to preliminary visual observations that a large portion of FCD depletion occurred within 0.1 mm from the lesions in these samples. Also, the analyzed regions away from lesions were chosen to be wide enough to get a reliable estimate of average FCD loss (%), and to reach approximately the same depth in tissue as the 0.1 mm region surrounding lesions. Subsequently, average FCD loss was calculated by comparing regional average optical density at day 7 or 12 to the day 0 regional average optical density. Taken together, in total we studied four different FCD analysis groups (freely-swollen, within 0.1 mm from lesion, away from lesion, and uninjured dynamically loaded) at three different timepoints (days 0, 7 and 12 after inducing mechanical injury).

**S1.3.1 Statistical analyses**

Data normality was assured with Anderson-Darling test for the average regional optical densities. One-way analysis of variance (ANOVA) was utilized to test for statistically significant changes in average optical densities between three groups at a time; between **(1)** freely-swollen disks, **(2)** within 0.1 mm from lesions, and **(3)** away from lesions at day 0, and between **(1)** freely-swollen disks, **(2)** uninjured dynamically loaded disks, and **(3)** away from lesions at days 0, 7 and 12. Furthermore, we used one-way ANOVA followed by Tukey’s honestly significant difference (HSD) *post hoc* test to account for optical density changes between different timepoints within all the four groups separately (that is, **(1)** freely-swollen samples, **(2)** injured dynamically loaded samples near lesions, **(3)** injured dynamically loaded samples away from lesions, and **(4)** uninjured dynamically loaded samples). For comparison of FCD loss between regions near and away from lesions in the same samples (at days 7 and 12 separately), the dependent samples *t*-test was utilized. With the level of significance set as α = 0.05, the statistical analyses were conducted with MATLAB (Statistics and Machine Learning Toolbox) and SPSS (version 25, IBM, Armonk, NY, USA).

**S1.4 Experimental combined biochemical and biomechanical degradation**

Li et al. [4] analyzed GAG loss with simultaneous inflammatory load (IL-6 (50 ng/ml), IL-6 soluble receptor (sIL-6R, 250 ng/ml), and TNFα (25 ng/ml) together, simulating inflamed tissue environment seen *in vivo* post-injury) and dynamical loading in injured cartilage samples. Cartilage disks were treated for eight days with (*n* = 18) and without (*n* = 18) initial mechanical injury.

**S1.5 Simulation of biochemical degradation**

In the biochemical model, the initial depth-dependent aggrecan distribution $C_{\text{agg,0}}$ in moles/m^3^ was [11,12]

$C_{\text{agg,0}}=-0.0433z^{4}+0.0431z^{3}-0.0162z^{2}+0.002z+0.0242$, (S1)

where *z* is the normalized distance from the cartilage bottom (*z* = 0) to the surface (*z* = 1). Due to the small pore size of cartilage caused partly by aggrecan-aggregates [13], the effective diffusion coefficients $D_{\text{eff,}\text{i}}$ of aggrecanases and cytokines IL-1 were modeled as aggrecan-dependent [12]

$D_{\text{eff,}\text{i}}=D_{i}\cdot\exp\left( -\lambda_{1,i}\cdot C_{\text{agg}} \right)$, (S2)

where $D_{i}$ is diffusivity of aforementioned chemical species *i*, $\lambda_{1,i}$ is a constant describing to what extent intact aggrecan affects the effective diffusivities [12] and $C_{\text{agg}}$ is aggrecan concentration. Therefore, the degradation of aggrecan content results in increased diffusion flux of more cytokines into tissue. Furthermore, even though the basal aggrecan synthesis rate *P*_agg_ (which implicitly included the passive diffusion of aggrecan out of the tissue (base degeneration rate) in the biochemical model) was constant, the total aggrecan production rate *R*_agg_ was depth-dependent and IL-1 concentration-dependent [12]

$R_{\text{agg}}=P_{\text{agg}}\cdot\left( 1-0.9z \right)\cdot\exp\left( -\lambda_{2}\cdot C_{\text{IL-1}} \right)$, (S3)

where $\lambda_{2}$ is a constant describing the inhibition of aggrecan synthesis by IL-1 and $C_{\text{IL-1}}$ is the IL-1 concentration. That is, the presence of pro-inflammatory cytokines modulated the aggrecan synthesis profile, partly leading to further FCD loss besides the degradative effects of ADAMTS.

For full description of the model and justification for its parameters, the readers are referred to the original article by Kar et al. [12]. Though not affecting changes of aggrecan concentration, we kindly note a small mistake in the model parameters, namely in the definition of aggrecan-dependent MMP catalytic activity *k*_act,mmp_. In the denominator, the exponent *n* (Hill-coefficient of MMP activity) should be -*n*. The activity coefficient should be of the form [14]

$\frac{\left[ L \right]^{n}}{\left[ L \right]^{n}+{K_{\text{A}}}^{n}}=\frac{1}{1+\left( \frac{K_{\text{A}}}{\left[ L \right]} \right)^{n}}$, (S4)

where [*L*] is the total ligand concentration (in Kar et al., this corresponds to sum of intact and degraded aggrecan concentrations, *C*_agg_+*C*_aggd_) and *K*_A_ is ligand concentration at which half the receptors are ligand-bound (in Kar et al., ligand-bound receptor is interpreted as increase in MMP catalytic activity, and *K*_A_ corresponds to intact aggrecan concentration at half-maximal MMP activity, *k*_9_). Therefore, aggrecan-dependent MMP catalytic activity should be

$k_{\text{act,mmp}}=\frac{\beta_{\text{max}}}{1+\left( \frac{C_{\text{agg}}+C_{\text{aggd}}}{k_{9}} \right)^{-n}}$, (S5)

where $\beta_{\text{max}}=1$ is the maximum MMP activity.

**S1.6 Data interpolation to COMSOL Multiphysics**

During each simulation iteration (50 in total), we ran one COMSOL and ABAQUS simulation. After these separate simulations, element-wise aggrecan loss (COMSOL) and FCD loss (ABAQUS) were extracted and combined to total FCD loss according to equations (3), (7) and (8) using MATLAB. Change in aggrecan content was treated as change in FCD content. For the next iteration, those FCD losses were updated to ABAQUS material files and to the COMSOL model. The latter (Eq. (4)) was done with the data interpolation tool of COMSOL. Before the interpolation, the algorithm has updated FCD values at the ABAQUS mesh, but not yet at the COMSOL mesh. To handle proper interpolation of aggrecan (and other chemical species) concentrations to the COMSOL mesh, an additional set of data points were set outside the ABAQUS mesh within 0.05 mm from the surfaces. Without these extra data points, data interpolation to COMSOL was problematic at the free surfaces (aggrecan values dropped close to zero). The extra data points were given the same values as nearest-neighbor ABAQUS mesh integration points. The COMSOL interpolation tool then used the data at the ABAQUS mesh integration points and element centroids augmented with the data set outside the ABAQUS mesh to perform interpolation to COMSOL mesh nodes, integration points and element centroids. The interpolation procedure caused 0.8% decrease in predicted FCD losses away from the lesion in the biochemical model where the aforementioned MATLAB-interface between the two software was established, compared to a biochemical model run only in COMSOL.

**S1.7 Biomechanical material model**

Cartilage was modeled as a fibril-reinforced porohyperelastic material with Donnan osmotic swelling and chemical expansion (FRPHES) [15–17] in ABAQUS. The FRPHES model captures well the experimental stress relaxation data of young calf cartilage in unconfined compression [6]. The same material model was used by Orozco et al. [18], who determined the initial depth-dependent FCD distribution according to depth-wise optical density profiles. All compositional and material parameters are presented in the Table S1. In brief, the constitutive model consists of fluid and solid phases, where the solid phase is divided into a fibrillar part and a swelling non-fibrillar part (PGs). For the non-fibrillar part, the Cauchy stress tensor of a neo-Hookean solid material is [15,19]

$$\boldsymbol{\sigma}_{\mathrm{nf}}=K_{\mathrm{nf}}\frac{\ln\left( J \right)}{J}\mathbf{I}+\frac{G_{\mathrm{nf}}}{J}\left( \mathbf{F}\cdot\mathbf{F}^{T}-J^{\frac{2}{3}} \mathbf{I} \right), \left( S6 \right)$$

where $\mathbf{F}$ is the deformation gradient tensor, $J$ = det($\mathbf{F}$) is volumetric deformation, and $\mathbf{I}$ is the unit tensor. $K_{\mathrm{nf}}$ and $G_{\mathrm{nf}}$ are the bulk and shear moduli of the non-fibrillar matrix, respectively,

$$K_{\mathrm{nf}}=\frac{E_{\mathrm{nf}}}{3\left( 1-2\nu_{\mathrm{nf}} \right)} , \left( S7 \right)$$

$$G_{\mathrm{nf}}=\frac{E_{\mathrm{nf}}}{2\left( 1+\nu_{\mathrm{nf}} \right)}, \left( S8 \right)$$

where $E_{\mathrm{nf}}$ and $\nu_{\mathrm{nf}}$ are the Young´s modulus and Poisson´s ratio of the non-fibrillar matrix, respectively.

The stress in collagen fibrils was modeled by a constant fibril network modulus $E_{f}$ [16]

$$\sigma_{f}=\left\{ \begin{matrix} E_{f}\varepsilon_{f}, & \varepsilon_{f}\geq0, \\ 0, & \varepsilon_{f}<0, \end{matrix} \right. \left( S9 \right)$$

where $\varepsilon_{f}$ is the logarithmic fibril strain ($\varepsilon_{f}$ = ln(||**F*e***_f,0_||, where ***e***_f,0_ is the unit vector of the initial fibril orientation). For a fibril *j* the Cauchy stress tensor is

$${\boldsymbol{\sigma}_{f}}^{j}=\left\{ \begin{matrix} \rho_{z}C\sigma_{f}\boldsymbol{e}_{f}\otimes\boldsymbol{e}_{f}, & for primary fibrils, \\ \rho_{z}\sigma_{f}\boldsymbol{e}_{f}\otimes\boldsymbol{e}_{f}, & for secondary fibrils, \end{matrix} \right. \left( S10 \right)$$

where $\rho_{z}$ is the depth-dependent collagen fraction per solid volume, $C$ is the density ratio between primary and secondary fibrils ($C$ = 3.009 [20]) and ⊗ symbolizes dyadic product. The 2D collagen network consisted of 2 primary fibrils defining curved basic structure found in young calf cartilage [10,20] and 7 secondary fibrils describing cross-links and randomly oriented fibrils [20]. $\boldsymbol{e}_{f}$ is the current normalized fibril orientation vector

$$\boldsymbol{e}_{f}=\frac{\mathbf{F}\boldsymbol{e}_{f,0}}{\left. \left| \left| \mathbf{F}\boldsymbol{e}_{f,0} \right| \right| \right.}. \left( S11 \right)$$

Fluid flow was incorporated according to Darcy’s law

$$q=-k\nabla p , \left( S12 \right)$$

where $q$ is the flow flux in the non-fibrillar matrix, $k$ is the hydraulic permeability and$\nabla p$ is the pressure gradient.

Donnan osmotic swelling pressure gradient in equilibrium is

$$\Delta\pi=\phi_{\mathrm{int}}RT\left( \sqrt{c_{F}^{2}+4\frac{\left( \gamma_{\mathrm{ext}}^{\pm} \right)^{2}}{\left( \gamma_{\mathrm{int}}^{\pm} \right)^{2}}c_{\mathrm{ext}}^{2}} \right)-2\phi_{\mathrm{ext}}RTc_{\mathrm{ext}}, \left( S13 \right)$$

where $c_{F}$ is the current depth-dependent FCD concentration, $\phi_{\mathrm{int}}$, $\phi_{\mathrm{ext}}$, $\gamma_{\mathrm{int}}^{\pm}$ and $\gamma_{\mathrm{ext}}^{\pm}$ are internal and external osmotic coefficients and internal and external activity coefficients, respectively [21], $c_{\mathrm{ext}}$ is the external salt concentration (0.15 M), *R* is the molar gas constant (8.314 J/mol K) and *T* is the absolute temperature (293.0 K). The chemical expansion stress is

$$T_{c}=a_{0}c_{F} \exp\left( -\kappa\frac{\gamma_{\mathrm{ext}}^{\pm}}{\gamma_{\mathrm{int}}^{\pm}}\sqrt{c^{-} \left( c^{-}+c_{F} \right)} \right), \left( S14 \right)$$

where $a_{0}$ and $\kappa$ are material constants [15] and $c^{-}$ is the mobile anion concentration. The current depth-dependent FCD concentration is modeled as a function of volumetric deformation

$$c_{\text{F}}=c_{\text{F,0}}\frac{n_{\text{f,0}}}{n_{\text{f,0}}-1+J} , \left( S15 \right)$$

where $c_{\text{F,0}}$ is the initial depth-dependent FCD and $n_{\text{f,0}}$ is the initial fluid volume fraction (*i.e.*, porosity). Finally, the total stress tensor is

$$\boldsymbol{\sigma}_{\mathrm{tot}}\boldsymbol{=}\sum_{j=1}^{totf} {\boldsymbol{\sigma}_{f}}^{j}+\boldsymbol{\sigma}_{\mathrm{nf}}\mathbf{-}\Delta\pi\mathbf{I-}T_{c}\mathbf{I-}\mu_{f}\mathbf{I}, \left( S16 \right)$$

where *totf* is the sum of primary and secondary fibrils (2 + 7 = 9 [20]) and $\mu_{f}$ is the chemical potential of water [22].

**Table S1.** Compositional and material parameters for the fibril-reinforced porohyperelastic swelling (FRPHES) material model. *z* is the normalized distance from the cartilage surface (*z* = 0) to the bottom (*z* = 1).

| **Parameter** | **Value** | **Description** | **Reference** |
| --- | --- | --- | --- |
| ***Compositional*** |  |  |  |
| $n_{f}$ (-) | $0.8-0.15z$ | Depth-dependent initial fluid fraction in equilibrium | [23] |
| $\rho_{z}$ (-) | $20.6z^{6}-64.4z^{5}+78.1z^{4}- 45.9z^{3}$  $+13.4z^{2} - 1.6z+ 0.96$ | Depth-dependent collagen fraction | [24] |
| $c_{F}$ (mEq/ml) | $-4.4z^{6}+15.2z^{5}-21.0z^{4}+ 14.9z^{3}$  $-5.8z^{2} + 1.1z+ 0.03$ | Depth-dependent fixed charge density | [18] |
| ***Material*** |  |  |  |
| *C* (-) | 3.009 | Ratio between primary and secondary collagen fibrils | [20] |
| $E_{f}$ (MPa) | 20.0 | Initial fibril network modulus | [18] |
| $E_{\mathrm{nf}}$ (MPa) | 0.16 | Non-fibrillar matrix modulus | [18] |
| $\nu_{\mathrm{nf}}$ (-) | 0.42 | Non-fibrillar matrix Poisson’s ratio | [25] |
| $k$ ($\text{m}^{\text{4}}\text{N}^{-1}\text{s}^{-1}\text{)}$ | $1.3\cdot{10}^{-15}$ | Hydraulic permeability | [18] |

**S1.8 Base degeneration term**

We included a “base degeneration term” *b* into the model which accounts for experimental FCD loss seen in freely-swollen samples due to passive diffusion of GAGs out of tissue. The parameter *b* was estimated from FCD losses in full-thickness 0.45 mm wide (±10%) slabs through the middle of disks at days 7 and 12. We included base degeneration to obtain comparable results with Kar et al. [12] who included this effect implicitly into their model in constant basal aggrecan production term and effective diffusivity of aggrecan. Iterative Gauss-Newton algorithm was used for parameter estimation according to a function estimating FCD loss

$$\mathrm{FC}D_{i\%}=\mathrm{FC}D_{0}\cdot\left( 1-b \right)^{i}, i=1,2,\ldots, 50, \left( S17 \right)$$

where $\mathrm{FC}D_{\text{i\%}}$ is the amount (%) of non-degraded FCD at iteration *i* and $\mathrm{FC}D_{0}$ is initial FCD concentration (100%). Gauss—Newton iteration (step size 0.1, 100 iterations, initial guess 0.02) provided an estimate of *b* = 0.0044. The base degeneration was thought to be happening in all the elements, similarly as Kar et al. [12] did, regardless of the biomechanical degeneration. Thus, the effects of base degeneration were treated as additive to that of biomechanical degradation, and the FCD loss was defined as

$\mathrm{FC}D_{\text{i}}=\mathrm{FC}D_{\text{i-}\text{1}}\cdot\left( 1-\left( D_{\text{mech}}+b \right) \right), i=1,2,\ldots, 50,$ (S18)

where $D_{\text{mech}}$ is the biomechanical degeneration rate (Eq. 6). With the base degeneration, the simulated FCD losses near the lesion corresponded better with the experiments compared to the biomechanical model without base degeneration (Fig 7B).

The loss of FCD in the absence of external loading or mechanical injury has also been seen experimentally [26,27]. Kar et al. [12] calibrated base degeneration rate to be 2% of bulk FCD loss over seven days of simulated *in vivo* conditions (diffusion allowed through top surface). Our *in vitro* estimations are slightly higher, 7% of bulk FCD loss in seven days (diffusion through top surface and sides). Base degeneration might be lower *in vivo* than *in vitro* since under physiological and healthy conditions the biochemical mediators likely promote aggrecan synthesis more compared to *in vitro* conditions [27]. This might compensate the *in vivo* FCD loss to synovial fluid. Furthermore, *in vitro* basal FCD loss could possibly be decreased with more physiological-like culture medium, as suggested by Durney et al. [27], thus making the base degeneration term *b* tend to zero in such a medium.

**S1.9 Mesh sensitivity**

The effect of mesh on the predicted FCD losses was tested with five meshes which were increasingly dense near the lesion and the free surfaces. Denser meshes predicted slightly elevated average FCD losses near the lesion and smaller FCD losses away from the lesion compared to coarser meshes (S1 Fig). The mesh with 918 elements was chosen for all the simulations, since increasing the mesh density beyond that resulted only in minor changes in predicted FCD losses near and away from the lesion.


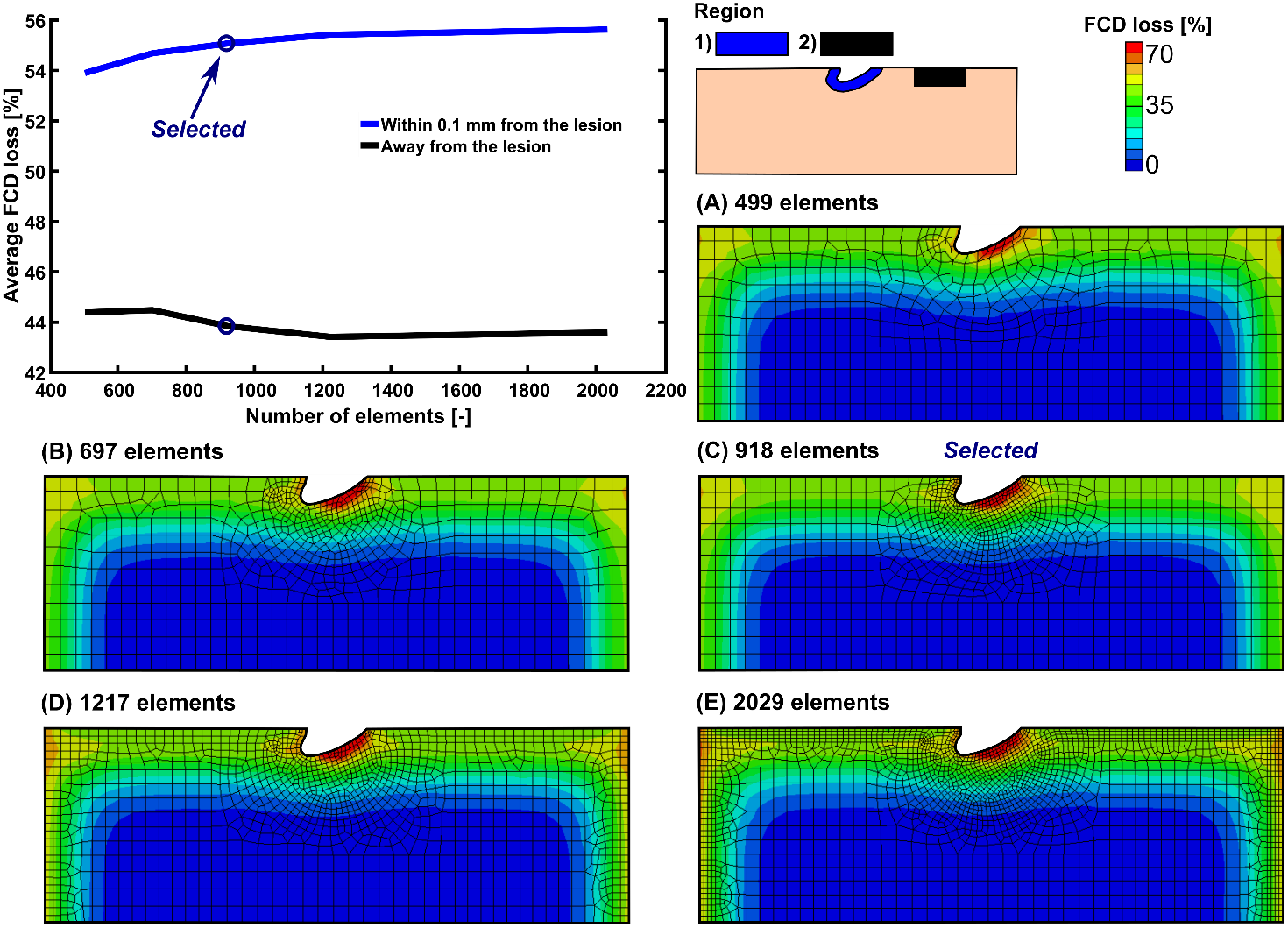


**S1 Fig**. Mesh sensitivity analysis. Average fixed charge density (FCD) loss at time *t* = 4 d with combined biochemical and biomechanical degradation with A) 499, B) 697, C) 918, D) 1217, and E) 2029 elements. The mesh with 918 elements was chosen, as increasing the mesh density from this did not yield quantitatively nor qualitatively different predictions for average FCD loss anymore.

**S2 Results**

**S2.1 Experiments**

Average optical density losses in biomechanical degradation experiments [6] were analyzed for freely-swollen, uninjured dynamically loaded and injured dynamically loaded disks at 0, 7 and 12 days after the start of experiment. At day 0, no significant differences (one-way ANOVA, *p* = 0.068) in average optical density between **(1)** freely-swollen disks, **(2)** injured disks away from lesions, and **(3)** injured disks near lesions were found (S2 Fig, panel A). Average optical densities decreased significantly over time in dynamically loaded injured samples both away (one-way ANOVA, *p* = 5.6$\cdot$10^-4^) and near (*p* = 2.6$\cdot$10^-7^) lesions with respect to day 0 (S2 Fig, panel B). Furthermore, average optical densities between **(1)** freely-swollen disks, **(2)** uninjured dynamically loaded disks, and **(3)** near lesions in injured dynamically loaded disks were similar at day 0 (one-way ANOVA, *p* = 0.105), day 7 (*p* = 0.213), and day 12 (*p* = 0.416) (S2 Fig, panel C). Within these three groups the average optical densities decreased over time; within the away from the lesion -group the decrease in average OD was significant (one-way ANOVA, *p* = 0.065 for freely-swollen, *p* = 0.075 for uninjured dynamically loaded, *p* = 5.6$\cdot$10^-4^ for injured dynamically loaded samples near lesions; S2 Fig, panel C). In injured samples, significantly more FCD loss was observed near lesions compared to regions away from lesions after both 7 (dependent samples *t*-test, *p* = 2.0$\cdot$10^-4^) and 12 (*p* = 9.9$\cdot$10^-3^) days of dynamic loading (S2 Fig, panel D).

**
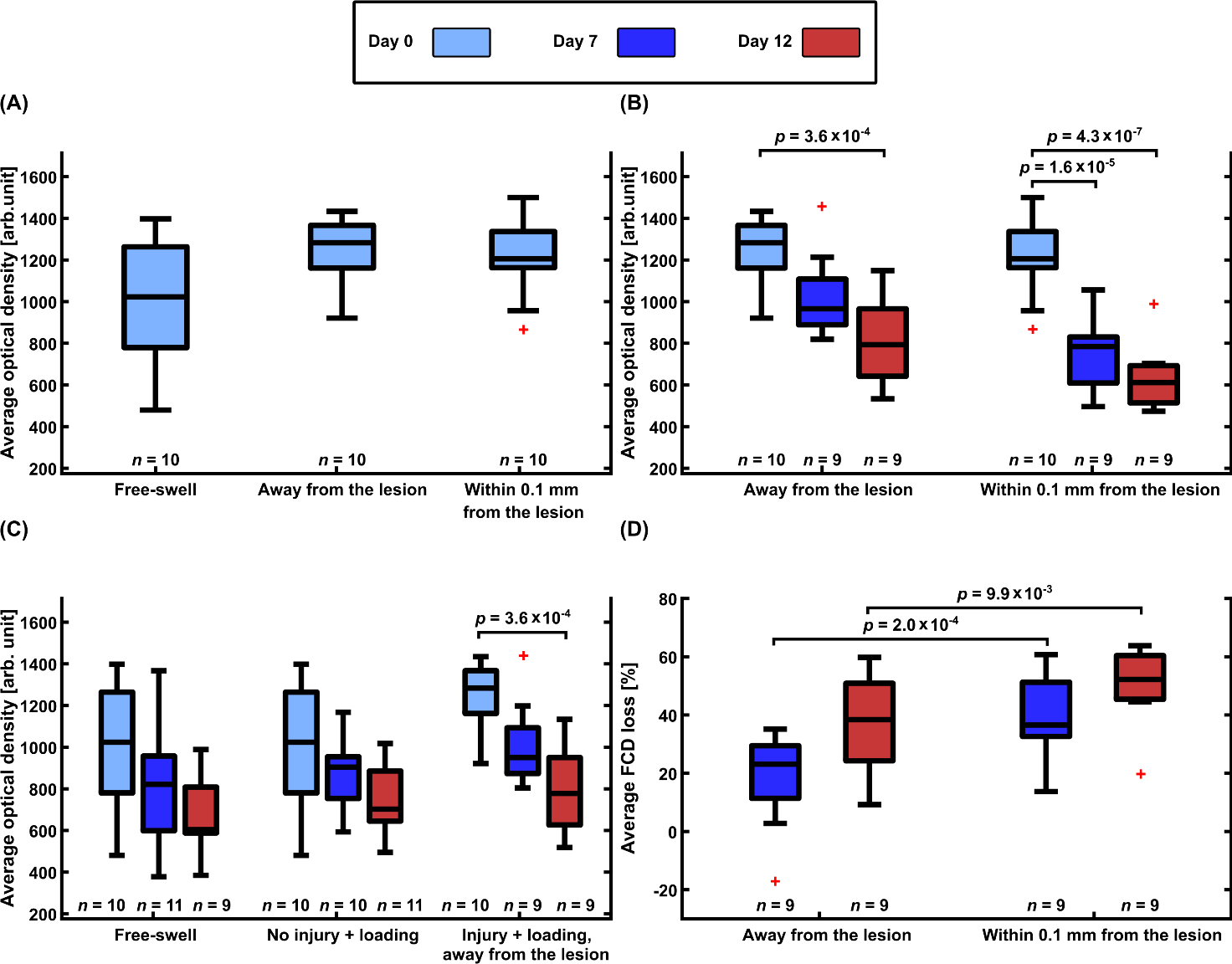
**

**S2 Fig:** Quantification of average optical density (OD) and fixed charge density (FCD) loss in freely-swollen control samples, uninjured dynamically loaded samples and injured dynamically loaded samples treated for 7 or 12 days. These biomechanical degradation experiments were carried out by Orozco et al. [6]. In injured samples, ODs were calculated as an average within 0.1 mm (±10%) from a lesion. In regions away from lesions, ODs were calculated as an average from a 0.45 mm (±10%) wide and 15 mm (±10%) thick surface region at the midway between lesion and sample edge. In freely-swollen and uninjured dynamically loaded samples, ODs were calculated as an average from a 0.45 mm (±10%) wide and 15 mm (±10%) thick surface region at the middle of samples. A) At day 0, average ODs between the freely-swollen and injured samples were similar (one-way ANOVA, *p* = 0.068). B) Average OD decreases significantly over time both away from lesions (one-way ANOVA, *p* = 5.6$\cdot$10^-4^) and near lesions (*p* = 2.6$\cdot$10^-7^; the figure shows Tukey's honestly significant difference (HSD) test results). C) At days 0, 7 and 12, average ODs were statistically similar between freely-swollen, uninjured dynamically loaded, and away from the lesion -groups (one-way ANOVA, *p* = 0.105 for day 0, *p* = 0.213 for day 7, *p* = 0.416 for day 12). However, within these treatment groups the average ODs decreased in time, especially in the away from the lesion -group which exhibited statistically significant decrease in OD (one-way ANOVA, *p* = 0.065 for freely-swollen samples, *p* = 0.075 for uninjured dynamically loaded samples, and *p* = 5.6$\cdot$10^-4^ for injured dynamically loaded samples away from lesions; the figure shows Tukey’s HSD test results). D) FCD losses (calculated from average ODs at day 7 and 12 compared to day 0) near lesions were significantly greater than away from lesions (the figure shows dependent samples *t*-test results). Box plots display values as range (brackets), interquartiles and median (solid bars).

**S2.2. Bulk loss of fixed charge density**

Average FCD loss in the whole geometry was highest in the combined biochemical and biomechanical degradation model (S3 Fig). The combined model and the biochemical degradation model exhibited similar sigmoidal shape in the FCD loss over time. Biomechanical model predicted relatively low average bulk FCD loss, indicating that the biomechanical degradation is very localized. Implementing the base degeneration increased the biomechanical FCD loss compared to biomechanical degradation model without base degeneration.


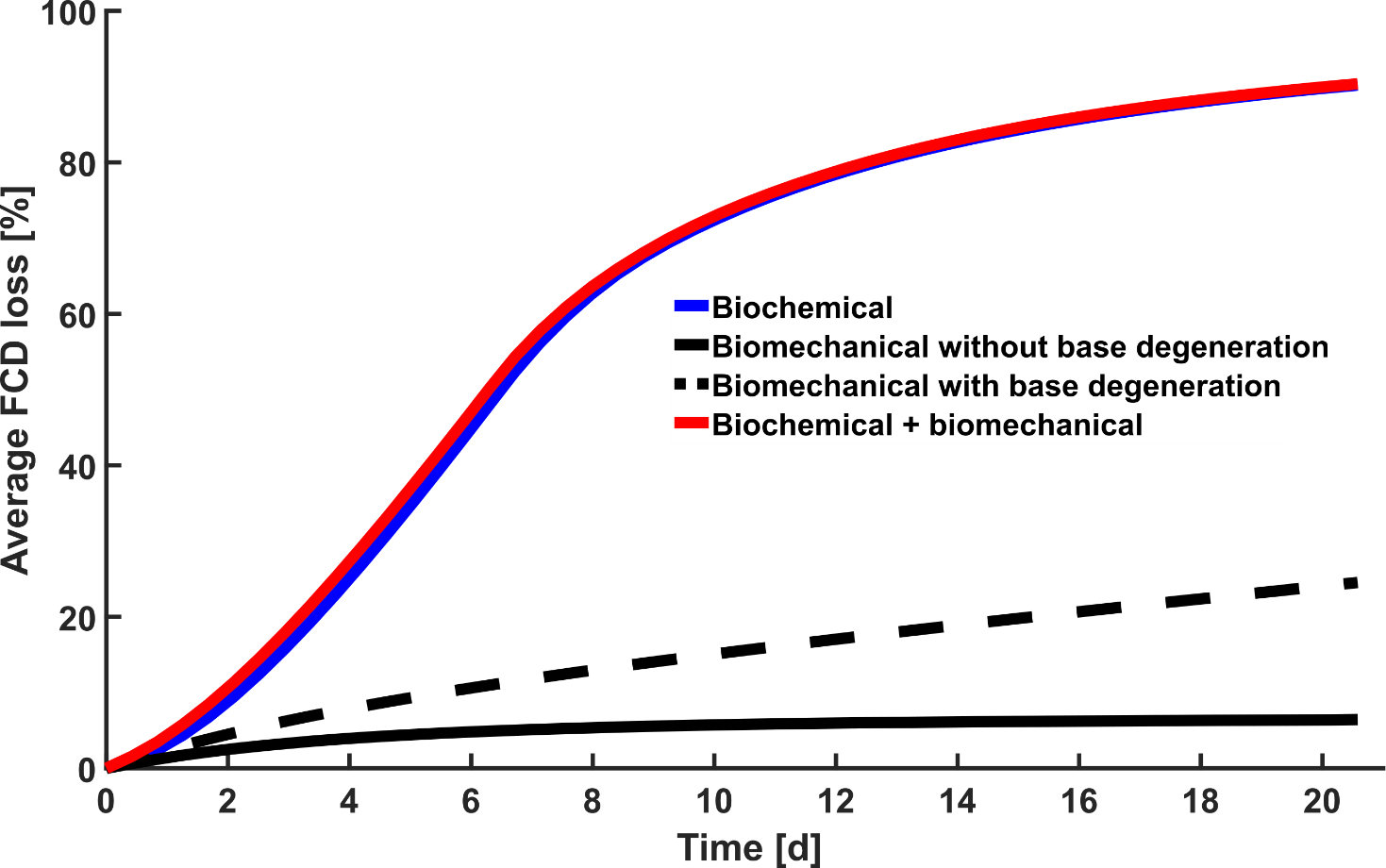
**S3 Fig**. Simulated average bulk fixed charge density (FCD) losses in the whole explant with biochemical, biomechanical (with and without base degeneration, see S1 Supplementary Material Subsection S1.8) and combined biochemical and biomechanical degradation models.

**S2.3 Physiological levels of cytokine interleukin-1**

The biochemical degradation models represented moderate inflammation. In order to compare cytokine-induced matrix losses between inflammatory state and physiological conditions, we modeled the FCD loss when the exogenous cytokine concentration was set to one hundredth of that in the original biochemical model (physiological 0.01 ng/ml vs moderate inflammation 1 ng/ml of IL-1)[12,28]. As expected, the model with 0.01 ng/ml of IL-1 predicted substantially lower FCD losses compared to model with 1 ng/ml of IL-1 (S4 Fig). As a sidenote, we also tested the biochemical model with 0 ng/ml of IL-1 (only passive diffusion present), which yielded 9.3% smaller bulk FCD losses at *t* = 4 d compared to 0.01 ng/ml model.

As Kar et al. [12] already demonstrated, also the matrix losses at physiological cytokine levels are in accordance with literature. Studying porcine cartilage, McNulty et al. [28] found a 350% increase in released sulphated glycosaminoglycan (sGAG) content after 24h of cytokine challenge with 1 ng/ml of IL-1α compared to 0.01 ng/ml. With the current model we predict a similar four-fold increase in FCD loss after 24h of culture away from the lesion with moderate inflammation compared to physiological conditions. For later timepoints, Lima et al. [29] reported 60% (day 14 of cytokine bath) and 70% (day 28) less retained GAG content in cell-seeded agarose constructs cultured with 1 ng/ml of IL-1α compared to 0.01 ng/ml. Our model with bovine cartilage predicts slightly more but comparable degradation away from the lesion (90% less retained FCD with inflammation compared to physiological conditions at day 14).


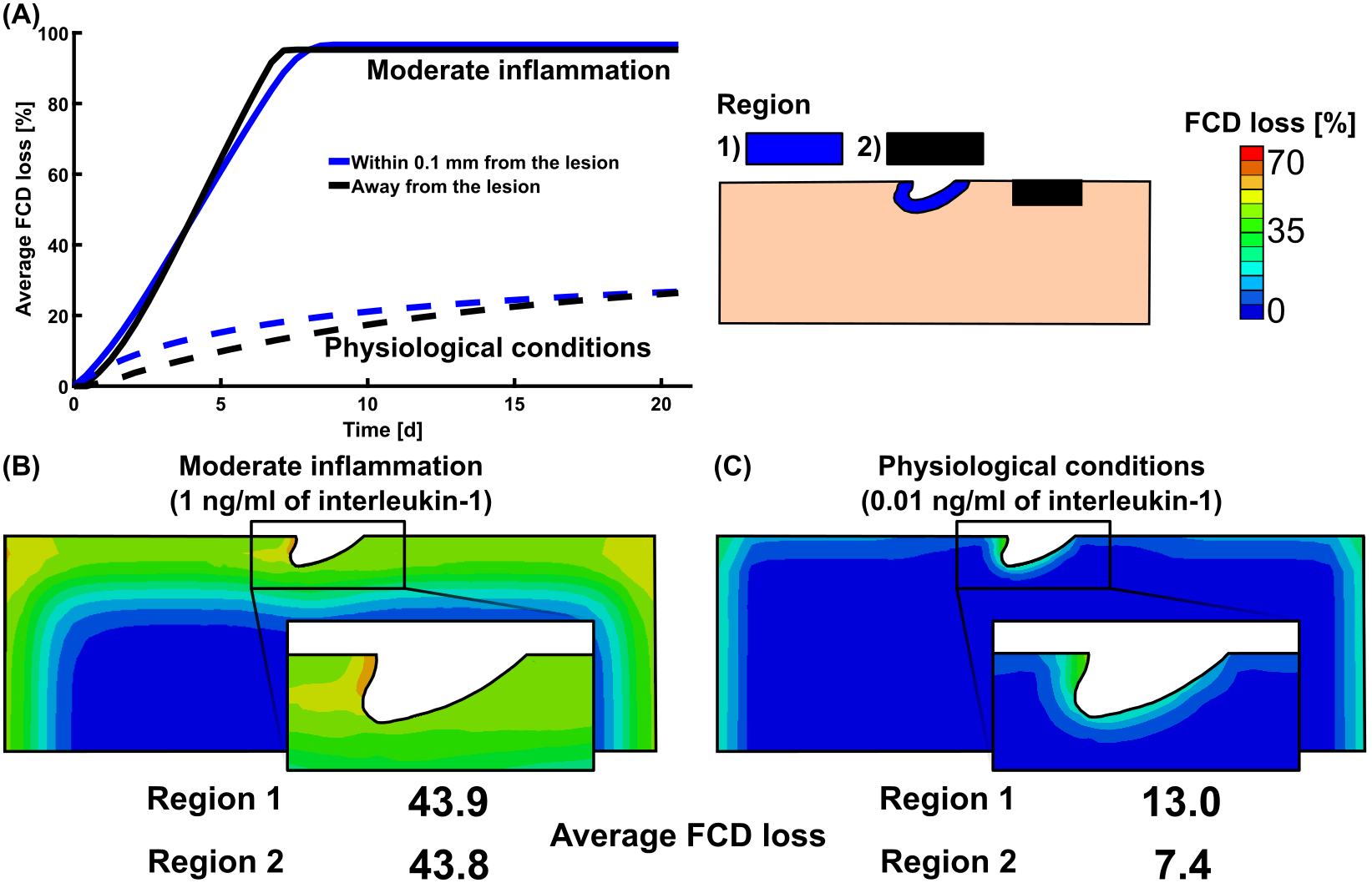


**S4 Fig**. A) Predicted biochemically driven fixed charge density (FCD) losses A) over time under moderate inflammation (1 ng/ml of exogenous interleukin-1) and physiological levels (0.01 ng/ml) of pro-inflammatory mediators. Still images at day *t* = 4 d show B) markedly higher matrix losses with moderate inflammation compared to C) physiological conditions.

**S2.4 Animations of predicted cartilage degeneration**

To show predicted damage progression in cartilage by our algorithm, we created animations showing the time-dependent degeneration (still images in S5 Fig). For visualization purposes only, cartilage was chosen to be “degraded” when average FCD loss in elements was equal or greater than 20%. The biochemical model animation (S1 Animation) shows an avalanche of FCD depletion originating from the free surfaces, whereas the biomechanical model predicts rapid FCD loss near the lesion during the first few days of dynamic loading (S2 and S3 Animation). In the combined biochemical and biomechanical degeneration animation, the biomechanical FCD loss occurs slightly earlier than the biochemical degradation near the lesion (S4 Animation).


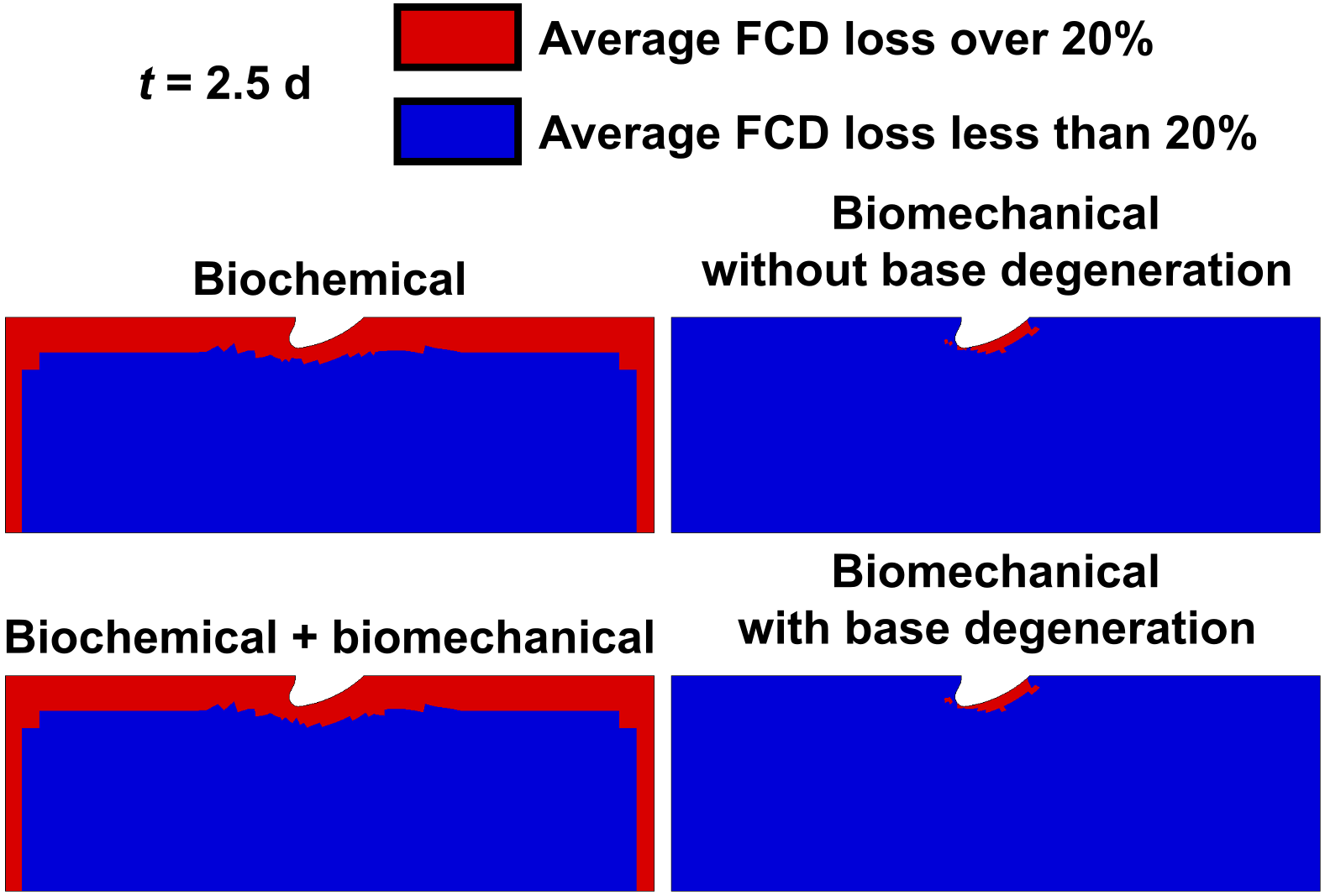


**S5 Fig**. Animations of predicted fixed charge density (FCD) losses over 21 days of biochemical, biomechanical (without or with base degeneration), and combined biochemical and biomechanical degradation.

**References**

[1] T.M. Quinn, A.A. Maung, A.J. Grodzinsky, E.B. Hunziker, J.D. Sandy, Physical and biological regulation of proteoglycan turnover around chondrocytes in cartilage explants. Implications for tissue degradation and repair, Ann. N. Y. Acad. Sci. 878 (1999) 420–441. doi:10.1111/j.1749-6632.1999.tb07700.x.

[2] P. Patwari, D.M. Cheng, A.A. Cole, K.E. Kuettner, A.J. Grodzinsky, Analysis of the relationship between peak stress and proteoglycan loss following injurious compression of human post-mortem knee and ankle cartilage, Biomech. Model. Mechanobiol. 6 (2007) 83–89. doi:10.1007/s10237-006-0037-y.

[3] A.L. Stevens, J.S. Wishnok, F.M. White, A.J. Grodzinsky, S.R. Tannenbaum, Mechanical Injury and Cytokines Cause Loss of Cartilage Integrity and Upregulate Proteins Associated with Catabolism, Immunity, Inflammation, and Repair, Mol. Cell. Proteomics. 8 (2009) 1475–1489. doi:10.1074/mcp.M800181-MCP200.

[4] Y. Li, E. Frank, Y. Wang, S. Chubinskaya, H.-H. Huang, A. Grodzinsky, Moderate Dynamic Compression Inhibits Pro-Catabolic Response of Cartilage to Mechanical Injury, TNF-α and IL-6, but Accentuates Degradation Above a Strain Threshold, Osteoarthr. Cartil. 21 (2013). doi:10.1016/j.joca.2013.08.021.

[5] Y. Li, Y. Wang, S. Chubinskaya, B. Schoeberl, E. Florine, P. Kopesky, A.J. Grodzinsky, Effects of insulin-like growth factor-1 and dexamethasone on cytokine-challenged cartilage: Relevance to post-traumatic osteoarthritis, Osteoarthr. Cartil. 23 (2015) 266–274. doi:10.1016/j.joca.2014.11.006.

[6] G.A. Orozco, P. Tanska, C. Florea, A.J. Grodzinsky, R.K. Korhonen, A novel mechanobiological model can predict how physiologically relevant dynamic loading causes proteoglycan loss in mechanically injured articular cartilage, Sci. Rep. 8 (2018) 1–16. doi:10.1038/s41598-018-33759-3.

[7] P. Patwari, G. Gao, J.H. Lee, A.J. Grodzinsky, J.D. Sandy, Analysis of ADAMTS4 and MT4-MMP indicates that both are involved in aggrecanolysis in interleukin-1-treated bovine cartilage, Osteoarthr. Cartil. 13 (2005) 269–277. doi:10.1016/j.joca.2004.10.023.

[8] B. Kurz, M. Jin, P. Patwari, D. Cheng, M. Lark, A. Grodzinsky, Biosynthetic response and mechanical properties of articular cartilage after injurious compression, J. Orthop. Res. 19 (2001) 1140–1146. doi:10.1016/S0736-0266(01)00033-X.

[9] M. DiMicco, P. Patwari, P. Siparsky, S. Kumar, M. Pratta, M. Lark, Y. Kim, A. Grodzinsky, Mechanisms and Kinetics of Glycosaminoglycan Release Following In Vitro Cartilage Injury, Arthritis Rheum. 50 (2004) 840–848. doi:10.1002/art.20101.

[10] B. Rolauffs, C. Muehleman, J. Li, B. Kurz, K. Kuettner, E. Frank, A. Grodzinsky, Vulnerability of the superficial zone of immature articular cartilage to compressive injury, Arthritis Rheum. 62 (2010) 3016–3027. doi:10.1002/art.27610.

[11] T.J. Klein, M. Chaudhry, W.C. Bae, R.L. Sah, Depth-dependent biomechanical and biochemical properties of fetal, newborn, and tissue-engineered articular cartilage, J. Biomech. 40 (2007) 182–190. doi:10.1016/j.jbiomech.2005.11.002.

[12] S. Kar, D.W. Smith, B.S. Gardiner, Y. Li, Y. Wang, A.J. Grodzinsky, Modeling IL-1 induced degradation of articular cartilage, Arch. Biochem. Biophys. 594 (2016) 37–53. doi:10.1016/j.abb.2016.02.008.

[13] M.A. DiMicco, R.L. Sah, Dependence of cartilage matrix composition on biosynthesis, diffusion, and reaction, Transp. Porous Media. 50 (2003) 57–73. doi:10.1023/A:1020677829069.

[14] R. Gesztelyi, J. Zsuga, A. Kemeny-Beke, B. Varga, B. Juhasz, A. Tosaki, The Hill equation and the origin of quantitative pharmacology, Arch. Hist. Exact Sci. 66 (2012) 427–438. doi:10.1007/s00407-012-0098-5.

[15] W. Wilson, C. van Donkelaar, B. van Rietbergen, R. Huiskes, A fibril-reinforced poroviscoelastic swelling model for articular cartilage, J. Biomech. 38 (2005) 1195–1204. doi:10.1016/j.jbiomech.2004.07.003.

[16] W. Wilson, C. van Donkelaar, B. van Rietbergen, K. Ito, R. Huiskes, Erratum to “Stresses in the local collagen network of articular cartilage: a poroviscoelastic fibril-reinforced finite element study” and “A fibril-reinforced poroviscoelastic swelling model for articular cartilage,” J. Biomech. 38 (2005) 2138–2140. doi:10.1016/j.jbiomech.2005.04.024.

[17] A. Eskelinen, M. Mononen, M. Venäläinen, R. Korhonen, P. Tanska, Maximum shear strain-based algorithm can predict proteoglycan loss in damaged articular cartilage, Biomech. Model. Mechanobiol. 18 (2019) 753–778. doi:10.1007/s10237-018-01113-1.

[18] G. Orozco, P. Tanska, C. Florea, A. Grodzinsky, R. Korhonen, A novel mechanobiological model can predict how physiologically relevant dynamic loading causes proteoglycan loss in mechanically injured articular cartilage, Sci. Rep. 8 (2018) 15599. doi:10.1038/s41598-018-33759-3.

[19] W. Wilson, J. Huyghe, C. van Donkelaar, Depth-dependent compressive equilibrium properties of articular cartilage explained by its composition, Biomech. Model. Mechanobiol. 6 (2007) 43–53. doi:10.1007/s10237-006-0044-z.

[20] W. Wilson, C.C. Van Donkelaar, B. Van Rietbergen, R. Huiskes, A fibril-reinforced poroviscoelastic swelling model for articular cartilage, J. Biomech. 38 (2005) 1195–1204. doi:10.1016/j.jbiomech.2004.07.003.

[21] J. Huyghe, G. Houben, M. Drost, C. van Donkelaar, An ionised/non-ionised dual porosity model of intervertebral disc tissue, Biomech. Model. Mechanobiol. 2 (2003) 3–19. doi:10.1007/s10237-002-0023-y.

[22] W. Wilson, C. van Donkelaar, J. Huyghe, A comparison between mechano-electrochemical and biphasic swelling theories for soft hydrated tissues., J. Biomech. Eng. 127 (2005) 158–165.

[23] V. Mow, X. Guo, Mechano-Electrochemical Properties Of Articular Cartilage: Their Inhomogeneities and Anisotropies, Annu. Rev. Biomed. Eng. 4 (2002) 175–209. doi:10.1146/annurev.bioeng.4.110701.120309.

[24] S. Saarakkala, P. Julkunen, Specificity of fourier transform infrared (FTIR) microspectroscopy to estimate depth-wise proteoglycan content in normal and osteoarthritic human articular cartilage, Cartilage. 1 (2010) 262–269. doi:10.1177/1947603510368689.

[25] L.P. Li, M.D. Buschmann, A. Shirazi-Adl, A fibril reinforced nonhomogeneous poroelastic model for articular cartilage: Inhomogeneous response in unconfined compression, J. Biomech. 33 (2000) 1533–1541. doi:10.1016/S0021-9290(00)00153-6.

[26] E.. Moo, N.. A. Osman, B. Pingguan-Murphy, The metabolic dynamics of cartilage explants over a long-term culture period, Clinics. 66 (2011) 1431–1436. doi:10.1590/s1807-59322011000800021.

[27] K.M. Durney, D. Sharifi Kia, T. Wang, A. Singh, L. Karbowski, H.J. Koo, G.A. Ateshian, M.B. Albro, Physiologic Medium Maintains the Homeostasis of Immature Bovine Articular Cartilage Explants in Long-Term Culture, J. Biomech. Eng. 141 (2018) 021004. doi:10.1115/1.4041901.

[28] A.L. McNulty, N.E. Rothfusz, H.A. Leddy, F. Guilak, Synovial fluid concentrations and relative potency of interleukin-1 alpha and beta in cartilage and meniscus degradation, J. Orthop. Res. 31 (2013) 1039–1045. doi:10.1002/jor.22334.

[29] E.G. Lima, A.R. Tan, T. Tai, L. Bian, G.A. Ateshian, J.L. Cook, C.T. Hung, Physiologic deformational loading does not counteract the catabolic effects of interleukin-1 in long-term culture of chondrocyte-seeded agarose constructs, J. Biomech. 41 (2008) 3253–3259. doi:10.1016/j.jbiomech.2008.06.015.
