# Supplementary figures and images for: Mechanobiological model for simulation of injured cartilage degradation via pro-inflammatory cytokines and mechanical stimulus
Source: PLoS Comput Biol. 2020 Jun 25;16(6):e1007998. doi: 10.1371/journal.pcbi.1007998 (PMC7343184; doi:10.1371/journal.pcbi.1007998)

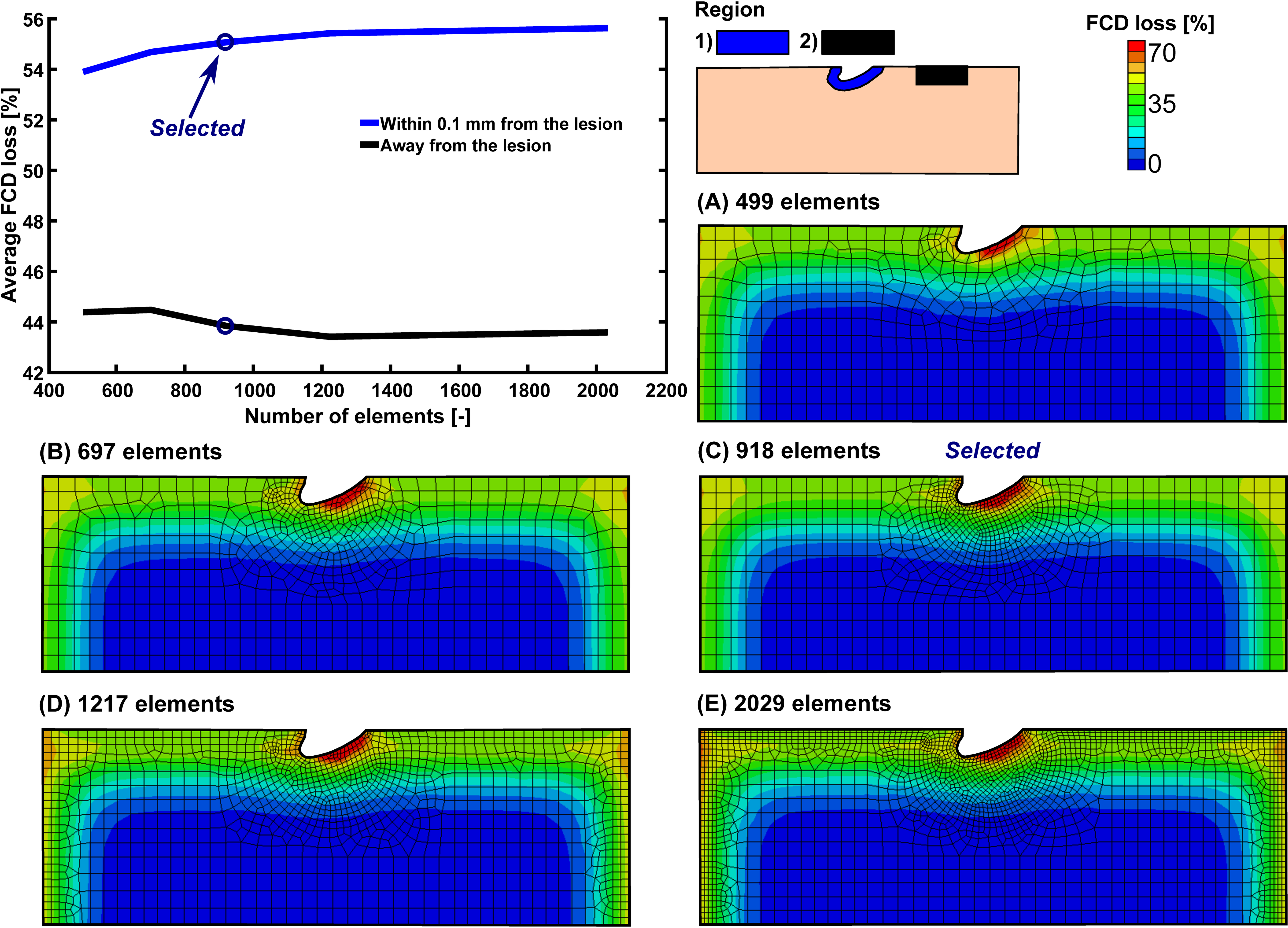

Supplement: S1 Fig — Average fixed charge density (FCD) loss at time t = 4 d with combined biochemical and biomechanical degradation with A) 499, B) 697, C) 918, D) 1217, and E) 2029 elements. The mesh with 918 elements was chosen, as increasing the mesh density from this did not yield quantitatively nor qualitatively different predictions for average FCD loss anymore. (TIF) [file pcbi.1007998.s002.tif]

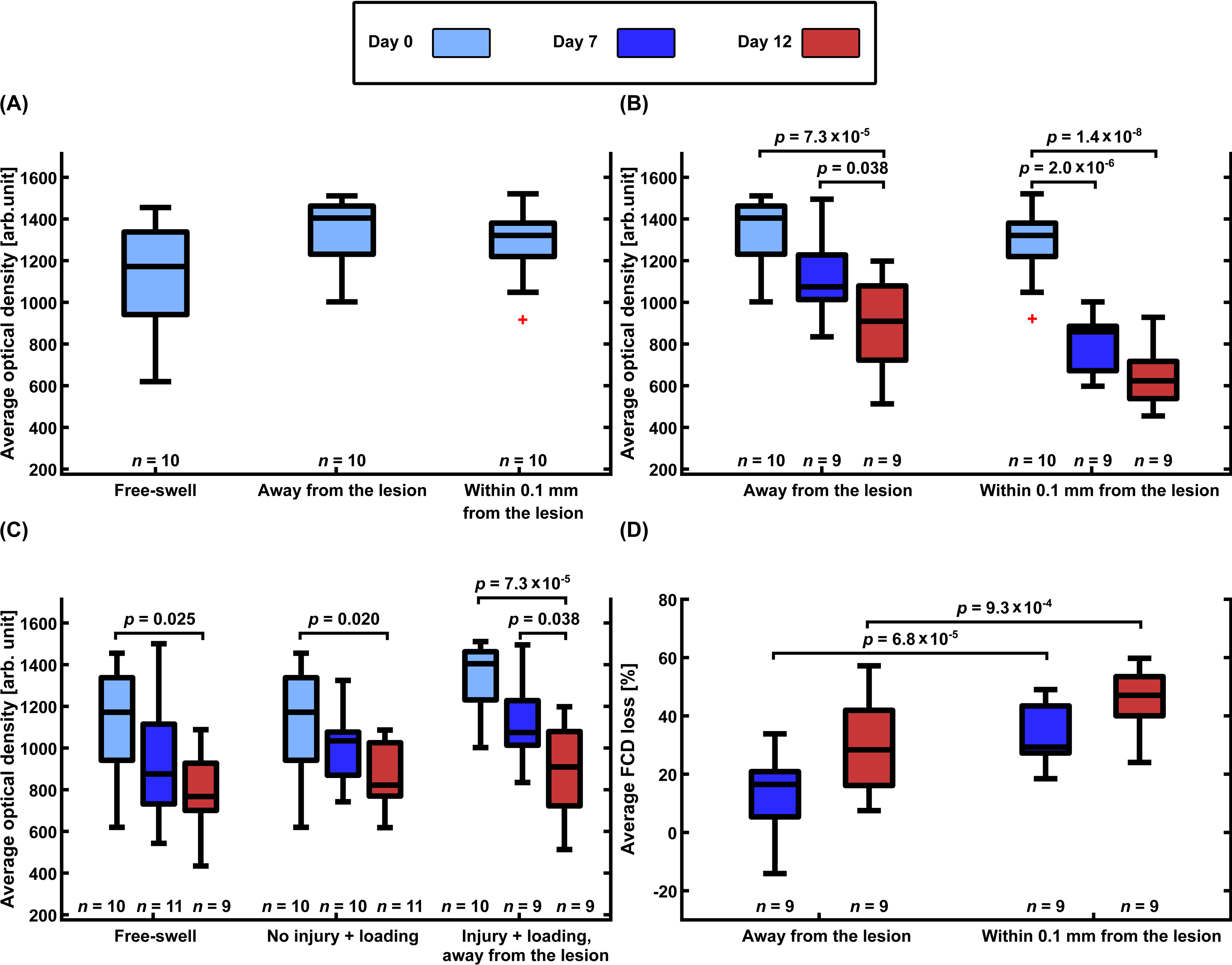

Supplement: S2 Fig — Quantification of average optical density (OD) and fixed charge density (FCD) loss in freely-swollen control samples, uninjured dynamically loaded samples and injured dynamically loaded samples treated for 7 or 12 days. These biomechanical degradation experiments were carried out by Orozco et al. [49]. In injured samples, ODs were calculated as an average within 0.1 mm (±10%) from a lesion. In regions away from lesions, ODs were calculated as an average from a 0.45 mm (±10%) wide and 15 mm (±10%) thick surface region at the midway between lesion and sample edge. In freely-swollen and uninjured dynamically loaded samples, ODs were calculated as an average from a 0.45 mm (±10%) wide and 15 mm (±10%) thick surface region at the middle of samples. A) At day 0, average ODs between the freely-swollen and injured samples were similar (one-way ANOVA, p = 0.068). B) Average OD decreases significantly over time both away from lesions (one-way ANOVA, p = 5.6⋅10−4) and near lesions (p = 2.6⋅10−7; the figure shows Tukey's honestly significant difference (HSD) test results). C) At days 0, 7 and 12, average ODs were statistically similar between freely-swollen, uninjured dynamically loaded, and away from the lesion -groups (one-way ANOVA, p = 0.105 for day 0, p = 0.213 for day 7, p = 0.416 for day 12). However, within these treatment groups the average ODs decreased in time, especially in the away from the lesion -group which exhibited statistically significant decrease in OD (one-way ANOVA, p = 0.065 for freely-swollen samples, p = 0.075 for uninjured dynamically loaded samples, and p = 5.6⋅10−4 for injured dynamically loaded samples away from lesions; the figure shows Tukey’s HSD test results). D) FCD losses (calculated from average ODs at day 7 and 12 compared to day 0) near lesions were significantly greater than away from lesions (the figure shows dependent samples t-test results). Box plots display values as range (brackets), interquartiles and median (solid bars). (TIF) [file pcbi.1007998.s003.tif]

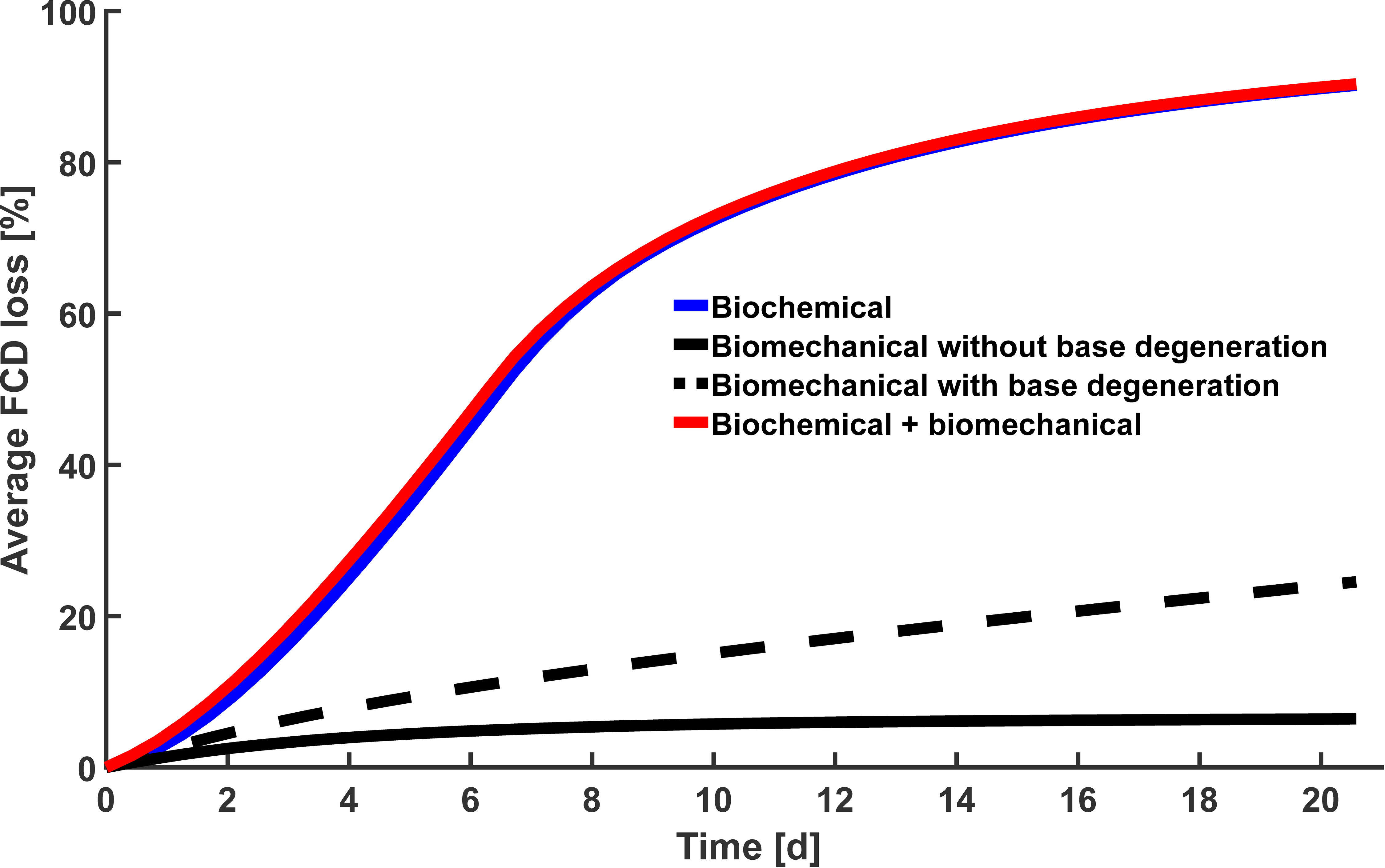

Supplement: S3 Fig — Simulated average bulk fixed charge density (FCD) losses in the whole explant with biochemical, biomechanical (with and without base degeneration, see S1 Supplementary Material Subsection S1.8) and combined biochemical and biomechanical degradation models. (TIF) [file pcbi.1007998.s004.tif]

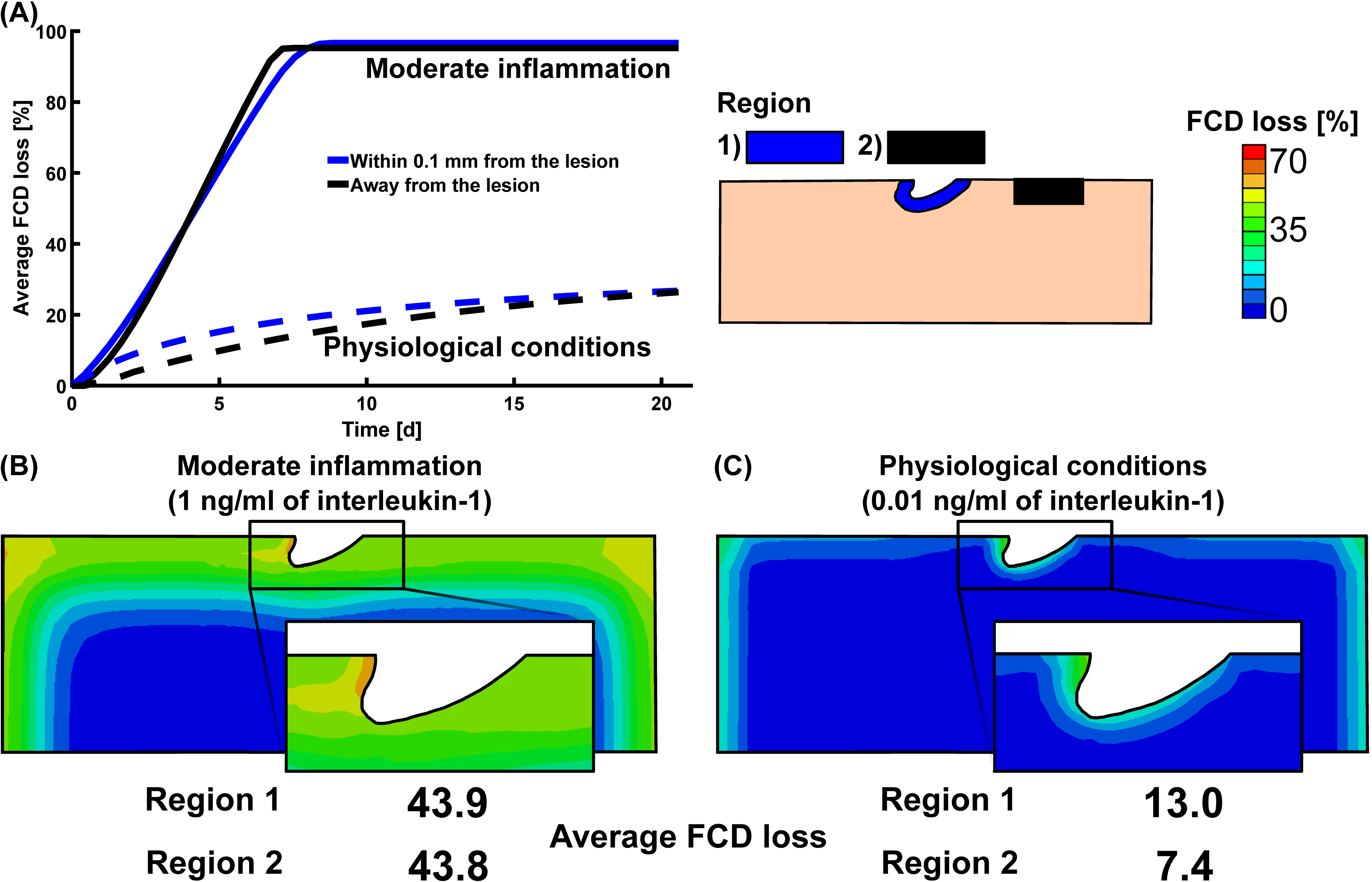

Supplement: S4 Fig — A) Predicted biochemically driven fixed charge density (FCD) losses A) over time under moderate inflammation (1 ng/ml of exogenous interleukin-1) and physiological levels (0.01 ng/ml) of pro-inflammatory mediators. Still images at day t = 4 d show B) markedly higher matrix losses with moderate inflammation compared to C) physiological conditions. (TIF) [file pcbi.1007998.s005.tif]

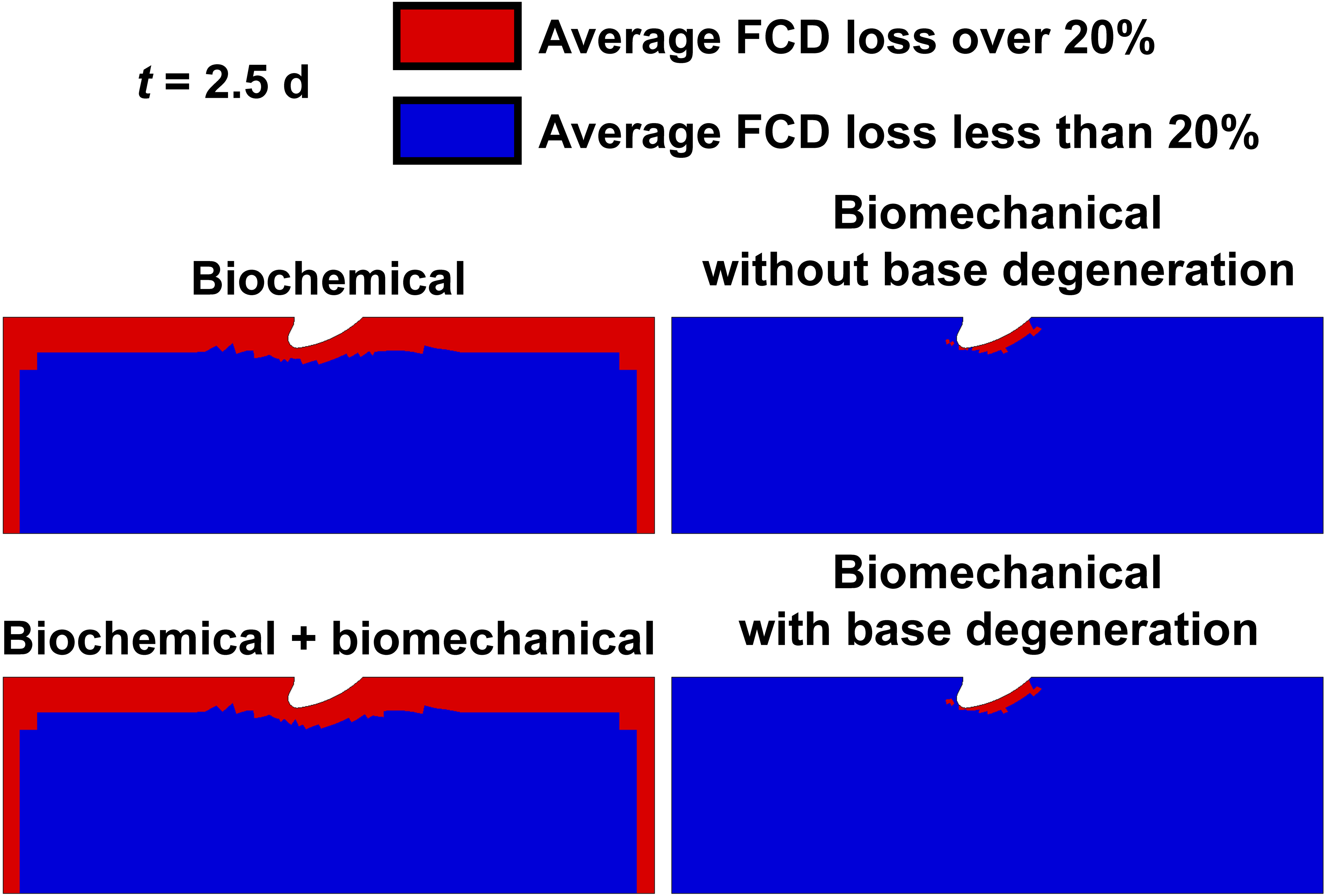

Supplement: S5 Fig — Animations of predicted fixed charge density (FCD) losses over 21 days of biochemical, biomechanical (without or with base degeneration), and combined biochemical and biomechanical degradation. (TIF) [file pcbi.1007998.s006.tif]

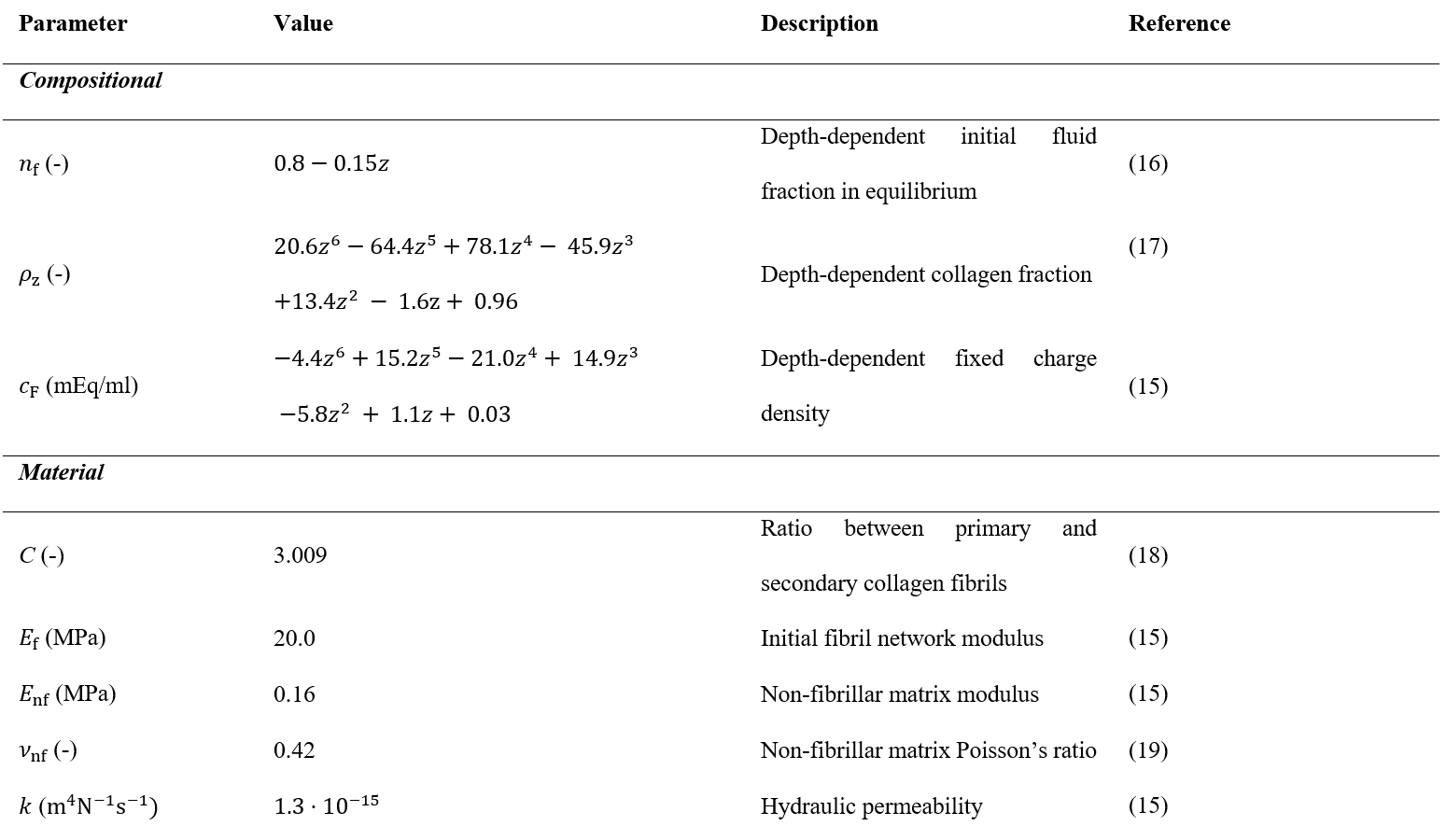

Supplement: S1 Table — z is the normalized distance from the cartilage surface (z = 0) to the bottom (z = 1). (PNG) [file pcbi.1007998.s007.PNG]

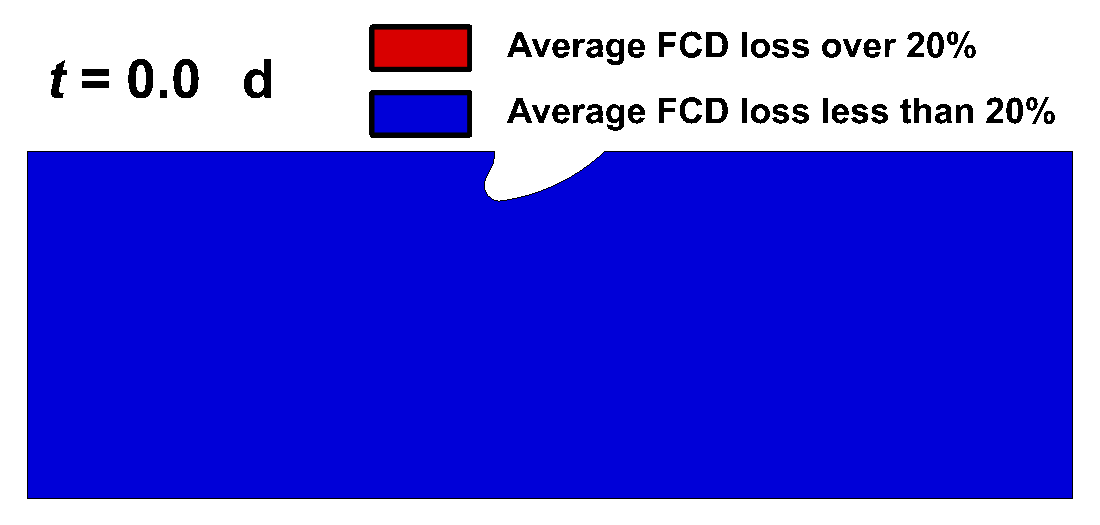

Supplement: S1 Animation — (GIF) [file pcbi.1007998.s008.gif]

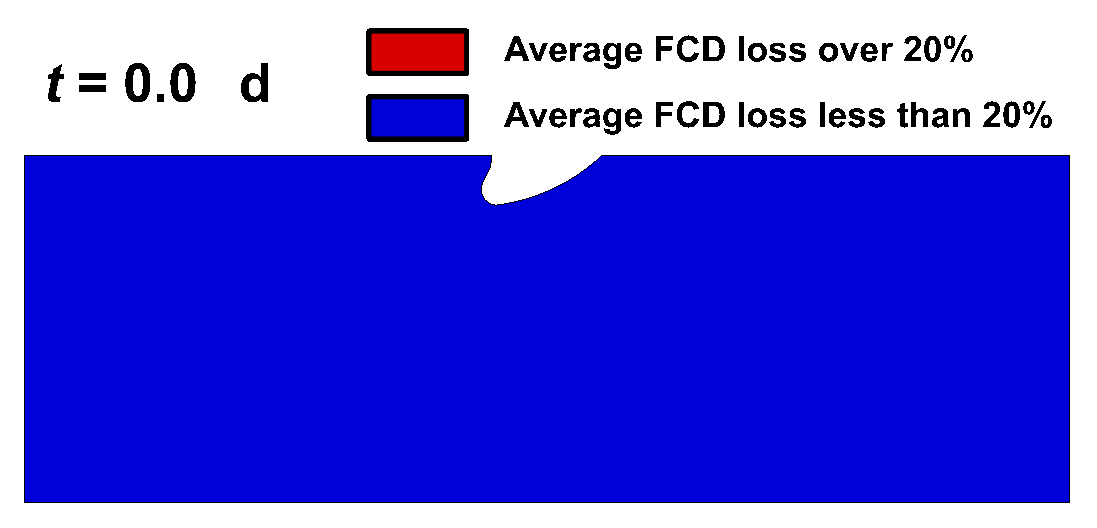

Supplement: S2 Animation — (GIF) [file pcbi.1007998.s009.gif]

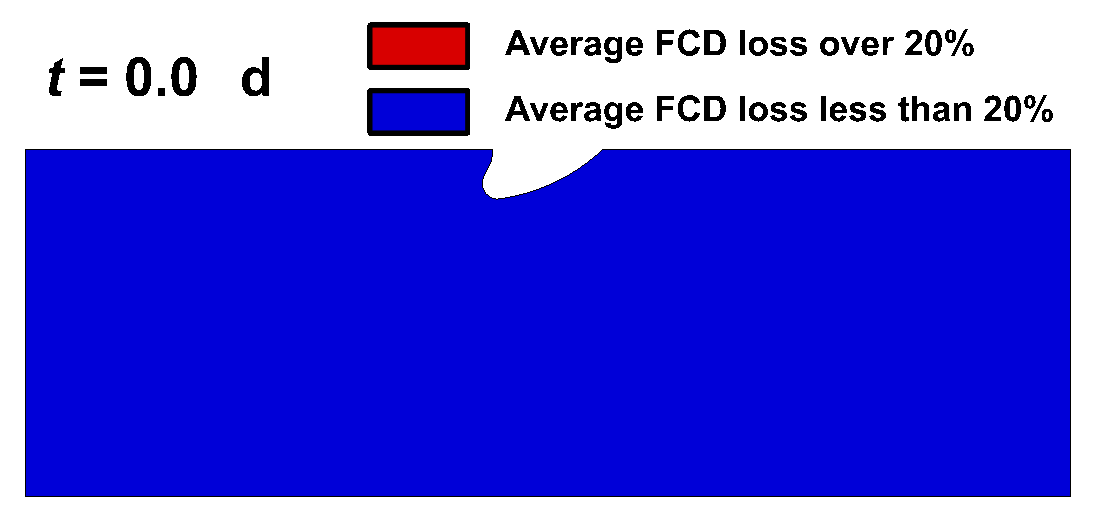

Supplement: S3 Animation — (GIF) [file pcbi.1007998.s010.gif]

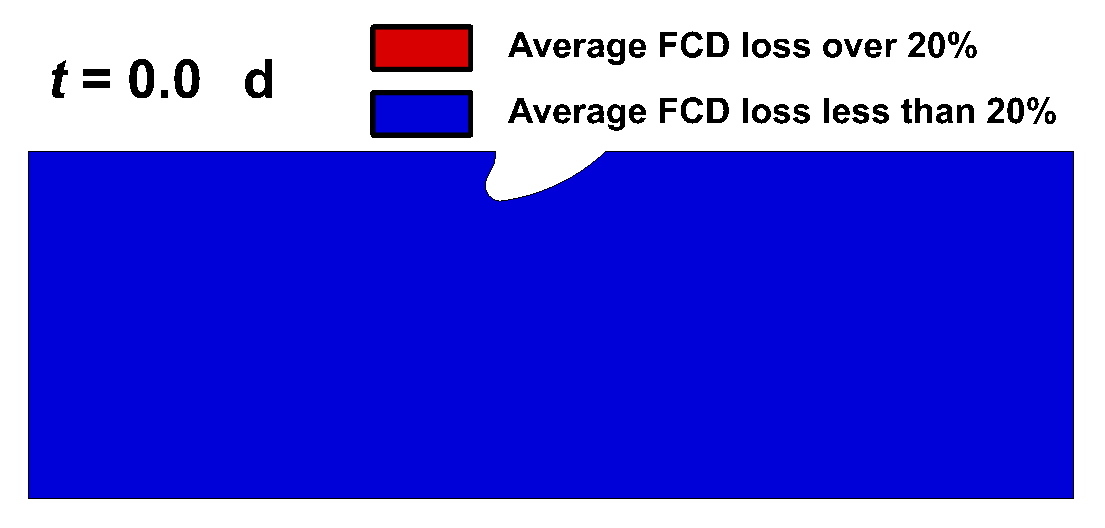

Supplement: S4 Animation — (GIF) [file pcbi.1007998.s011.gif]
