# Supplementary material for: Mechanobiological model for simulation of injured cartilage degradation via pro-inflammatory cytokines and mechanical stimulus
Source: PLoS Comput Biol. 2020 Jun 25;16(6):e1007998. doi: 10.1371/journal.pcbi.1007998 (PMC7343184; doi:10.1371/journal.pcbi.1007998)
Supplement: Attachment — Submitted filename: Eskelinen_responses_final.docx [file pcbi.1007998.s012.docx]

**Mechanobiological model for simulation of injured cartilage degradation via pro-inflammatory cytokines and mechanical stimulus**

Atte S.A. Eskelinen^1^, Petri Tanska^1^, Cristina Florea^1,2^, Gustavo A. Orozco^1^, Petro Julkunen^1,3^,

Alan J. Grodzinsky^2^ and Rami K. Korhonen^1^

^1^*Department of Applied Physics, University of Eastern Finland, Yliopistonranta 1 F, 70211 Kuopio, Finland*

^2^*Departments of Biological Engineering, Electrical Engineering and Computer Science and Mechanical Engineering, Massachusetts Institute of Technology, 77 Massachusetts Avenue, Cambridge, MA 02139, USA*

^3^*Department of Clinical Neurophysiology, Kuopio University Hospital, Puijonlaaksontie 2, 70210 Kuopio, Finland*

***RESPONSES TO THE REVIEWERS´ COMMENTS:***

The Authors express their deepest gratitude to the Reviewers for the considerable time and effort taken in the review of our manuscript. We appreciate your constructive comments and recommendations for the improvement of the document. We addressed all your comments and believe that we now have a much better manuscript.

**REVIEWER #1:** This paper presents a new finite element model to predict the simultaneous effect of degrading mechanisms on FCD content. This model incorporates diffusion of pro-inflammatory cytokine interleukin-1 into tissue and the effect of excessive levels of shear strain near chondral defects during physiologically relevant loading. Comsol, Abaqus and Matlab are used together to implement this complicated computational model. Good agreements with experiments and literature results are obtained.

**AUTHOR RESPONSE:** We thank the Reviewer for the praises. No changes to the manuscript were done based on these supportive comments.

**REVIEWER #2:** Summary: Eskelinen et al. describe a novel finite element model to simulate spatial and temporal changes of FCD content in injured cartilage. The model incorporates (1) diffusion of the proinflammatory cytokine interleukin-1 into tissue, and (2) the effect of excessive levels of shear strain near chondral defects. They suggest that the presence of cartilage lesions plays a role in cytokine diffusion-driven degradation, and predisposes cartilage for further biomechanical degradation.

Osteoarthritis (OA) is an important disease which affects many people and a complete therapy still remains elusive. Therefore, further basic and translational research studies focusing on underlying mechanism of pathogenesis and potential diagnostic strategies are needed. Thus, the manuscript would contribute towards solving an unmet clinical need.

The manuscript is very well and comprehensively written including copious and detailed additional information and rationales in the supplementary. The abstract is straightforward and adequate. The introduction includes all needed information and gives an overview on the disease and pathogenesis as well as the clinical need. The M&M and the results section are clearly and understandable written.

The discussion is critical, all important limitations have been mentioned, and includes extensive literature search.

**AUTHOR RESPONSE:** The Authors appreciate the Reviewer’s compliments. In the following, we go through the specific comments and suggested changes by the Reviewer. The parts that we have changed in the revised manuscript are marked with a yellow background.

***INTRODUCTION***

**REVIEWER COMMENT:** line 76 "on the other hand" makes no sense here

**AUTHOR RESPONSE AND ACTION:** (Page 5, line 91) Thank you for pointing this out, “on the other hand” was removed as redundant.

**REVIEWER COMMENT:** line 78/79 cartilage ECM - better to say ECM of cartilage

**AUTHOR RESPONSE AND ACTION:** (Page 5, line 93) The Authors thank the Reviewer for this suggestion which makes the sentence clearer. The wording is now “… signaling cascades that promote degradation of the extracellular matrix (ECM) of cartilage”.

**REVIEWER COMMENT:** line 79/80 Cytokines originate primarily from cells in synovial lining, and they can recruit more cytokines to the inflamed area – wrong wording – additional cells/immune cells are recruited to the area + produce more cytokines - cytokines itself can’t be recruited

**AUTHOR RESPONSE AND ACTION:** (Page 5, lines 94-96) Thank you for noting this mistake in the text. The sentences were modified as follows: “Cytokines originate primarily from cells in the synovial lining; subsequent increase in cytokine signaling can lead to recruitment of additional immune cells to the inflamed area, and the newly recruited cells produce even more cytokines”.

**REVIEWER COMMENT:** line 81 to specific chondrocyte (cartilage cell) receptors – better to say: cell receptors on chondrocyte surface

**AUTHOR RESPONSE AND ACTION:** (Page 5, line 97): The wording was changed: “… bind to cell receptors on chondrocyte (cartilage cell) surface”.

**REVIEWER COMMENT:** line 101 what does tissue stresses means?

**AUTHOR RESPONSE AND ACTION:** (Page 6, lines 117-118) With tissue stress we mean the mechanical stress (ratio of force and area) experienced by tissues when they are subjected to mechanical loading. To further clarify this, the wording was changed to “… the effects of mechanical loading-modulated tissue stresses (such as maximum principal stress) …”.

***MATERIALS AND METHODS***

**REVIEWER COMMENT:** Paragraph 2.2. The authors have implemented the Kar model in COMSOL Multiphysics, this code enables the simultaneous solution of PDEs and ODEs (which both occurs in the Kar model). The biomechanical part, i.e. the model of Orozco et al. has been modelled with ABAQUS. Thus, the main idea of the paper is to combine both models. As described also in the paper of Orozco the FCD loss has been computed using an iterative scheme implemented in MATLAB. Even after reading the original paper of Orozco it remains unclear whether this is done for each ABAQUS (fixed) grid point separately and how the number of iterations corresponds to the number of 21 days (as noted in line 238).

**AUTHOR RESPONSE:** Thank you for the excellent comment, further clarification is certainly needed. In the model of this paper, the biomechanical tissue response was calculated at each integration point of each finite element. The decision of 21 days in COMSOL matching 50 iterations in ABAQUS was based on several assumptions and findings: **(1)** 21 days was used by Kar et al. for the model calibration, **(2)** 50 iterations was found to result in near-equilibrium in terms of biomechanical FCD loss, displaying < 0.15% FCD loss progression after the iteration 40, **(3)** setting 21 days to correspond to 50 iterations led to FCD loss predictions that matched reasonably well with the experiments done by us (Fig. 7B) and found from the literature (Rolauffs *et al*. (2010), Thibault *et al*. (2002), DiMicco *et al*. (2004), Mohanraj *et al*. (2014), McNulty *et al*. (2013)). In this model, the timeline is linear, *i.e.* each iteration corresponds to 21/50 d $\approx$ 10 h of simulated time; the fixed timepoints (1*21/50 d, 2*21/50 d, 3*21/50 d, and so forth) were the same in both COMSOL Multiphysics and ABAQUS. In essence, both software ran 50 consecutive simulations each simulating a time period of 21/50 d with updating the FCD content between iterations, as clarified now in Subsections 2.2.4. and 2.2.8. Therefore, the degeneration equations for the combined model embedded with 21 days and 50 iterations are phenomenological equations with which we attempt to predict FCD loss from scarce experimental data.

However, we acknowledge that more experimental data with different temporal endpoints is needed in order to better calibrate the timescale of the model. It might also not be a real-life scenario that the (biomechanical) FCD loss reaches an equilibrium state; for example, FCD loss might lead to higher fluid velocities, altered biomechanical degeneration threshold, or crack propagation during dynamic loading which in turn may lead to further accelerated FCD loss.

**AUTHOR ACTION:** (Materials and Methods, page 12, lines 258-259) Clarify Subsection 2.2.4. “50 iterations in total corresponded to 21 days of simulated time, so each iteration simulated a time period of 21/50 d $\approx$ 10 h; see more details from Subsection 2.2.8”

(Materials and Methods, page 15, lines 325-332) Clarify Subsection 2.2.8. “Simulation time of 21 days in COMSOL was chosen to correspond to 50 iterations in ABAQUS. 21 days was used originally for the biochemical model calibration (10) and 50 iterations were found to result in near-equilibrium of biomechanically-driven FCD loss (negligible change in FCD loss with more iterations, see Subsection 3.1.2). To further justify our decision, the biomechanical degeneration rate (Eq. 6) (17,51) combined with this time scale and the number of iterations captures well the FCD loss near lesions in our experiments (see Subsection 3.1.2 and S1 Supplementary Material Subsection S2.1) and the rapid early loading-associated GAG loss in mechanically loaded samples (83,86–88) (see Subsection 4.3).”

(Results, page 18, lines 389-390) “… the predicted FCD distribution had reached near-equilibrium (Fig. 7B, solid blue line; the FCD loss changed less than 0.15% per iteration after iteration 40)”

(Discussion, page 27, lines 582-586) Added a paragraph: “The biomechanical model without the base degeneration reached a near-equilibrium state in terms of FCD loss. We acknowledge that this might not be a real-life scenario; for example, FCD loss might lead to higher fluid velocity (49), altered shear strain threshold ($\hat{\varepsilon}_{\mathrm{threshold}}$ in Eq. (6)), or crack propagation during dynamic loading which in turn might lead to accelerated FCD loss. However, the model with the selected assumptions led to consistent results with the experiments.”

**REVIEWER COMMENT:** Furthermore, it should be mentioned that the Kar model is solved by a time integration, whereby the biomechanical model solves a stationary problem (if the reviewer has understood the whole methodology correctly).

**AUTHOR RESPONSE:** Thank you for noting this. The biochemical model was indeed solved by time integration. The biomechanical model solved a highly non-linear problem 50 times consecutively using a transient SOILS analysis in ABAQUS (non-static analysis) which considers the viscoelastic effects of the chosen material model (fluid flow) and the dynamic loading-unloading boundary condition.

**AUTHOR ACTION:** (Page 10, lines 204-206) “Biochemically-driven aggrecan loss was modeled via diffusion of IL-1 cytokines (1 ng/ml in the culture medium) into tissue and subsequent increase in protease biomolecule concentrations (time integration in COMSOL Multiphysics).”

(Page 13, lines 276-280) “The biomechanical model solved a highly non-linear problem consecutively (50 times, updating FCD content between iterations) with a transient SOILS analysis in ABAQUS to account for the viscoelastic behavior of the fibril-reinforced porohyperelastic material with Donnan osmotic swelling and chemical expansion (see S1 Supplementary Material Subsection S1.7 for further details on the material model).”

**REVIEWER COMMENT:** The variable MMP in the Kar model is obviously identical to ADAMTS in the manuscript, this should be mentioned and explained why it is here named differently. In paragraph 2.2.2 some parts of the Kar model are presented using indices j1 and j2. Using j as general index and indices 1 and 2 as specific indices would be certainly clearer. If there are differences to the published model of Kar they should be mentioned in each case or the whole set of equations should be presented, but only in the supplement.

**AUTHOR RESPONSE AND ACTION:** Thank you for the comment, the notation was indeed confusing. We changed the notation for stimulus equation for ADAMTS throughout the manuscript from $S_{j_{2}}$ to $S_{1}$ to be consistent with Kar et al. Also, we changed $C_{\text{IL-1-IL-1R-complex}}$ to be $C^{*}$ like in Kar et al., and index *j*_1_ to be *j*.

**REVIEWER COMMENT:** Most readers of the manuscript will have severe difficulties to understand paragraphs 2.2.6 to 2.2.8, to be honest also the reviewer. Perhaps it could be a good idea, to completely move the detailed description of the mechanical model (which can be similarly found in the paper of Orozco) into the supplement and to reduce the content of these paragraphs to a clear description of the input and the output of the approach without expecting detailed knowledge in elastomechanics.
The authors could also decide to give only a very short overview of the methodology, like it was done for the Kar model. Both would significantly shorten the length of the manuscript, even if the already extensive supplement could then be further extended.

**AUTHOR RESPONSE AND ACTION:** Thank you for the excellent idea of moving some of the material about the biomechanical model to the Supplementary Material. We decided to move only the subsection describing the material model to the Supplementary Material, since we prefer to keep the description of boundary conditions and main equations (*i.e.*, how FCD was decreased) in the revised manuscript in order to have a proper explanation of our approach and avoid confusing the readers. Now, the manuscript explains only the inputs, outputs, boundary conditions, and the most important equations for both biochemical (pages 10-13) and biomechanical model (pages 13-14). The numbering of the subsections and equations and referring to them were modified accordingly.

***DISCUSSION***

**REVIEWER COMMENT:** In the discussion all results of the simulations were carefully and probably exhaustively compared to a number of experimental findings. Also, several limitations of the approach were discussed in detail. For example, most of the large number of parameters within the Kar model are only "calibrated" but not validated by a parameter optimization including a sensitivity analysis. Thus, the manuscript shows, as promised in the abstract, that a simultaneous simulation of mechanical and biochemical processes is possible, even if there is still a long way to a validated full model for the processes during PTOA or, as a further example, rheumatic arthritis. There is also a strong demand for programming environments which can simultaneously perform simulations for interconnected biochemical and mechanical (and other) processes, without the tedious coupling of separate solvers for parts of the whole system of equations. To demonstrate this difficulty, the reviewer would recommend to present some examples for the computing times.

**AUTHOR RESPONSE:** Good points. We agree with the Reviewer's opinion. For the biochemical simulation, one COMSOL Multiphysics simulation (21 days) alone takes approximately two minutes, but when coupled with the biomechanical ABAQUS simulation (50 iterations) the time taken is approximately three days due to the complex material model. Based on this comment, we have included some lines in the revised manuscript.

**AUTHOR ACTION:** (Page 28, lines 612-617) Added a paragraph: “The current study also demonstrates the high need for fast and robust programming environments or software which could handle both inflammatory and biomechanical aspects simultaneously. This would reduce the strenuous coupling of several solvers and make the approach less complex and faster. Currently, the COMSOL Multiphysics can run the biochemical simulation alone in two minutes, but due to the complex biomechanical material model in ABAQUS the full simulation with both degradation mechanisms takes three days.”

**REVIEWER COMMENT:** For the reviewer and probably also for prospective readers, a list of abbreviations would be extremely helpful.

**AUTHOR RESPONSE AND ACTION:** Excellent suggestion. A list of abbreviations was added.

(Introduction, Page 7, line 143) Added sentence: “See Table 1 for a list of abbreviations”.

(Introduction, Page 7, line 144) New table:

**Table 1. List of abbreviations.**

| **Abbreviation** | **Description** |
| --- | --- |
| 2D/3D | 2-dimensional/3-dimensional |
| ACL | anterior cruciate ligament |
| ADAMTS | a-disintegrin and metalloproteinase with thrombospondin motifs |
| DD | digital densitometry |
| DMMB | dimethylmethylene blue |
| ECM | extracellular matrix |
| FCD | fixed charge density |
| FE | finite element |
| FRPHES | fibril-reinforced porohyperelastic swelling |
| GAG | glycosaminoglycan |
| IL | interleukin |
| MMP | matrix metalloproteinase |
| OD | optical density |
| PG | proteoglycan |
| PTOA | post-traumatic osteoarthritis |
| sIL-6R | interleukin-6 soluble receptor |
| TGF-β | transforming growth factor beta |
| TIMP | tissue inhibitors of metalloproteinases |
| TNFα | tumor necrosis factor alpha |

Additionally, the Author Summary was added.

(Page 3, lines 50-64) “**Author Summary [now 159 words]**

Post-traumatic osteoarthritis is a musculoskeletal disorder where inflammatory processes and abnormal joint loading predispose articular cartilage to degradation after a mechanical injury. Since inflamed and injured cartilage cannot be reversed back to healthy state, prevention of osteoarthritis progression is advisable, a prestigious goal where computational models could serve as tools. The current literature is short of computational models combining both biochemical and biomechanical aspects of osteoarthritis. Thus, here we implemented inflammation of living cartilage tissue followed by biochemical perturbations of tissue homeostasis and shear strain-induced biomechanical degradation in novel cell-to-tissue-level finite element models. The models presented in this paper and enriched by our experimental findings/previous literature provide profound new mechanobiological insights and predictions about cartilage degradation in injured and inflamed tissue under physiologically relevant mechanical loading. We suggest that mechanobiological computational models could be applied as in silico analysis tools that provide clinicians information of the personalized progression of post-traumatic osteoarthritis and decision-making guidance for treatment of the disease.”
